# Doping Up the Light: A Review of A/B-Site Doping in Metal Halide Perovskite Nanocrystals for Next-Generation LEDs

**DOI:** 10.1021/acs.jpcc.4c00749

**Published:** 2024-06-06

**Authors:** Ying Lu, Firoz Alam, Javad Shamsi, Mojtaba Abdi-Jalebi

**Affiliations:** † Institute for Materials Discovery, University College London, Malet Place, London WC1E 7JE, United Kingdom; ‡ Department of Electronic and Electrical Engineering, University College London, London WC1E 6BT, United Kingdom; § Cavendish Laboratory, Department of Physics, University of Cambridge, Cambridge CB3 0HE, United Kingdom

## Abstract

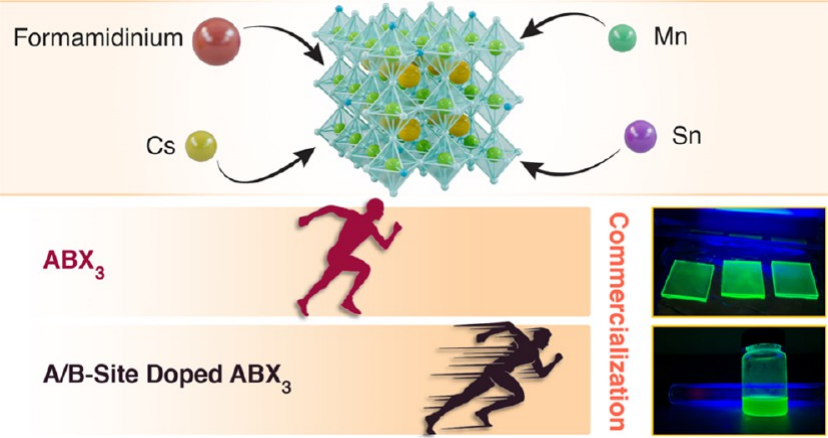

All-inorganic metal halide perovskite nanocrystals (PeNCs) show great potential for the next generation of perovskite light-emitting diodes (PeLEDs). However, trap-assisted recombination negatively impacts the optoelectronic properties of PeNCs and prevents their widespread adoption for commercial exploitation. To mitigate trap-assisted recombination and further enhance the external quantum efficiency of PeLEDs, A/B-site doping has been widely investigated to tune the bandgap of PeNCs. The bandgap of PeNCs is adjustable within a small range (no more than 0.1 eV) by A-site cation doping, resulting in changes in the bond length of Pb–X and the angle of [PbX_6_]^4^. Nevertheless, B-site doping of PeNCs has a more significant impact on the bandgap level through modification of surface defect states. In this perspective, we delve into the synthesis of PeNCs with A/B-site doping and their impacts on the structural and optoelectronic properties, as well as their impacts on the performance of subsequent PeLEDs. Furthermore, we explore the A-site and B-site doping mechanisms and the impact of device architecture on doped PeNCs to maximize the performance and stability of PeLEDs. This work presents a comprehensive overview of the studies on A-site and B-site doping in PeNCs and approaches to unlock their full potential in the next generation of LEDs.

## 1 Introduction

**Figure 1:**
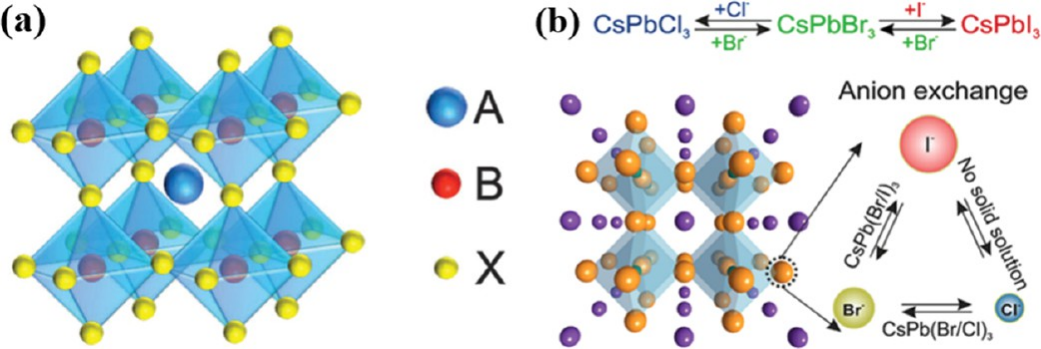
(a) Structure of perovskite ABX_3_^2^ and (b) schematic of anion exchange.^2^ Reproduced with permission from ref (2). Copyright 2016 Royal Society of Chemistry.

The excellent optical and electronic properties of lead-halide perovskite nanocrystals (LHP NCs) render them attractive building blocks for the development of next-generation optoelectronic devices, making them an area of intense research activity. The inherent tunability of bandgaps, high photoluminescence efficiency, and exceptional color purity have established them as promising candidates for the fabrication of high-performance LEDs. These desirable properties of LHP NCs have been attributed to their ability to efficiently confine excitons within their crystalline lattices, leading to a high radiative recombination rate. The perovskite crystal lattice is a complex network of BX_6_ octahedra that are arranged in a corner-sharing configuration.^1^ This arrangement forms the fundamental building block of perovskite structures, which are characterized by a general ABX_3_ stoichiometry, as depicted in Figure 1a.^2^ As a result of the efficient vacancy-assisted diffusion, halide anions within LHP NCs exhibit high charge carrier mobility, facilitating their facile extraction and substitution with different halides. In 2015, Kovalenko and colleagues were the pioneers in showcasing the rapid achievement of anion exchange at room temperature, as depicted in Figure 1b. This breakthrough allowed for easy tuning of bandgap energies and PL spectra throughout the entire visible range, spanning from 410 to 700 nm.^3^

LHP nanocrystals have emerged as promising materials for the production of LEDs. Within the realm of enhancing the performance of lead-halide perovskite LEDs (PeLEDs), the incorporation of A-site and B-site doping has displayed significant potential in the manipulation of their bandgap and ionic composition. Numerous studies have substantiated that A-site and B-site doping represent effective strategies for bolstering both the stability and the photoluminescence quantum yield (PLQY) of these devices. Although the impact of dopants has been demonstrated in the optical and structural properties of nanocrystals, it still needs to be carefully studied in the performance of lighting devices. As an example, the precise impact of these dopants on the stability and operational characteristics of these devices remains uncertain. Furthermore, whether these dopants introduce adverse effects on conventional perovskite devices, including LEDs, solar cells, and detectors, remains an area of ambiguity.

LHP NCs can be broadly categorized into two groups based on the nature of the A-site constituent. The first category consists of organic and inorganic cations, where the A-site is occupied by an organic cation, such as CH_3_NH_3_^+^ and HC(CH_2_)_2_^+^. The second category includes only inorganic metallic cations where the A-site is occupied by Rb^+^ and/or Cs^+^. Organic group cations in mixed LHP NCs are highly susceptible to environmental factors, such as oxygen, moisture, and temperature. As a result, their delicate crystal structure can easily break down, severely limiting their potential applications in optoelectronics.^4,5^^4,5^^4,5^^4,5^^4,5^ To overcome this constraint, it is imperative to study alternative LHP NCs that exhibit greater stability and are less prone to decomposition. All-inorganic LHP NCs, have garnered considerable attention in recent years due to their heightened thermal stability resulting from the use of inorganic cations, which possess higher thermal decomposition temperatures.^6,7^

Despite the swift implementation of LHP NCs in practical applications, the body of research on their synthesis continues to expand, with a growing number of synthesis protocols distinguished by factors such as precursor composition, ligand chemistry, solvents, NC surface treatments, and other postsynthetic processing steps.^8−19^ In essence, the motivation driving these intensive synthesis endeavors is rooted in the obstacles that arise from the LHP NCs’ intrinsically fragile and unstable nature. Among various approaches to improve the structural and optical stabilities of LHP NCs. A-site and B-site doping has demonstrated significant promise in fine-tuning the bandgap and ionic composition of these materials. While various studies have indicated that A-site and B-site doping can enhance the optoelectronic properties of LHP NCs, such as improving their PLQY and optical stability, this area of research is still in its early stages. The precise influence of these dopants on the stability and performance of perovskite lighting devices remains unclear. Moreover, whether these dopants have adverse effects on conventional perovskite devices such as solar cells, LEDs, and detectors is yet to be determined.

Halide PeLEDs share a manufacturing process similar to organic LEDs (OLEDs) for achieving large-area light emission and offer high luminous efficiency similar to inorganic LEDs.^20,21^ Halide PeLEDs have low exciton binding energies due to their fabrication at low temperatures. This results in luminescence behavior in halide PeLEDs at room temperature that is not dominated by excitons (almost no excitonic quenching), but rather by bandgap excitation luminescence.^22−25^ Halide PeLEDs can maintain high luminous quantum efficiency even at very high luminous intensity, which means they can achieve high-efficiency light emission at high brightness levels.^20^ The first halide PeLEDs were successfully fabricated by Wang et al. in 2014. The external quantum efficiency (EQE) increased significantly with an increasing current density. This is because the light-emitting principle of halide PeLEDs is different from that of OLEDs, as they do not heavily rely on exciton behavior for light emission.^23,24^ Moreover, the halide PeLEDs lights up at low voltage, and once lit, the current density increases significantly with increasing voltage, mainly because of the enhanced electron mobility at higher voltages.^20^ The EQE of the halide PeLEDs produced in 2018 reached 20.7% in the near-infrared (NIR) range, which is comparable to the EQE achieved by the best OLEDs of that year.^26^ Therefore, halide PeLEDs have great potential for use in various light-emitting devices due to their high efficiency and other advantageous properties.

To maximize the electroluminescence efficiency of perovskite-based LEDs, Kim et al. developed a one-dopant alloying strategy. This strategy reduces nonradiative recombination, enhances radiative recombination, suppresses defects, and improves charge carrier confinement. They applied this approach to the formamidinium lead bromide (FAPbBr_3_) system doped with a zero-dipole guanidinium cation (CH_6_N^3+^; GA^+^). The result was the highest electroluminescence efficiency observed in perovskite-based LEDs, with a current efficiency of 108 cd A^–1^ and an EQE of 23.4%, as well as a CE of 203 cd A^–1^ and an EQE of 45.5%. These efficiencies are comparable to the highest current efficiencies of conventional III–V and II–VI inorganic quantum-dot LEDs.

## 2 Synthesis Methodology

The literature widely acknowledges that hot-injection methods enjoy popularity due to their ability to provide precise control over various aspects of nanocrystal synthesis. These include size (which influences emission color), size distribution (affecting photoluminescence line width), shape, composition, and surface passivation (impacting PLQY). This control is achieved through the manipulation of synthesis conditions, such as reaction time, temperature, choice of solvent, precursor types, and their concentrations. Surface ligands employed during the synthesis of nanocrystals play a pivotal role in fine-tuning their properties, including processability, reactivity, and stability. These ligands are essential for preventing agglomeration and promoting dispersion in a range of solvents. However, it is crucial to note that improper selection of ligands can adversely impact the performance of optoelectronic devices. Therefore, the careful and appropriate choice of ligands is of the utmost importance in nanocrystal synthesis.

**Figure 2:**
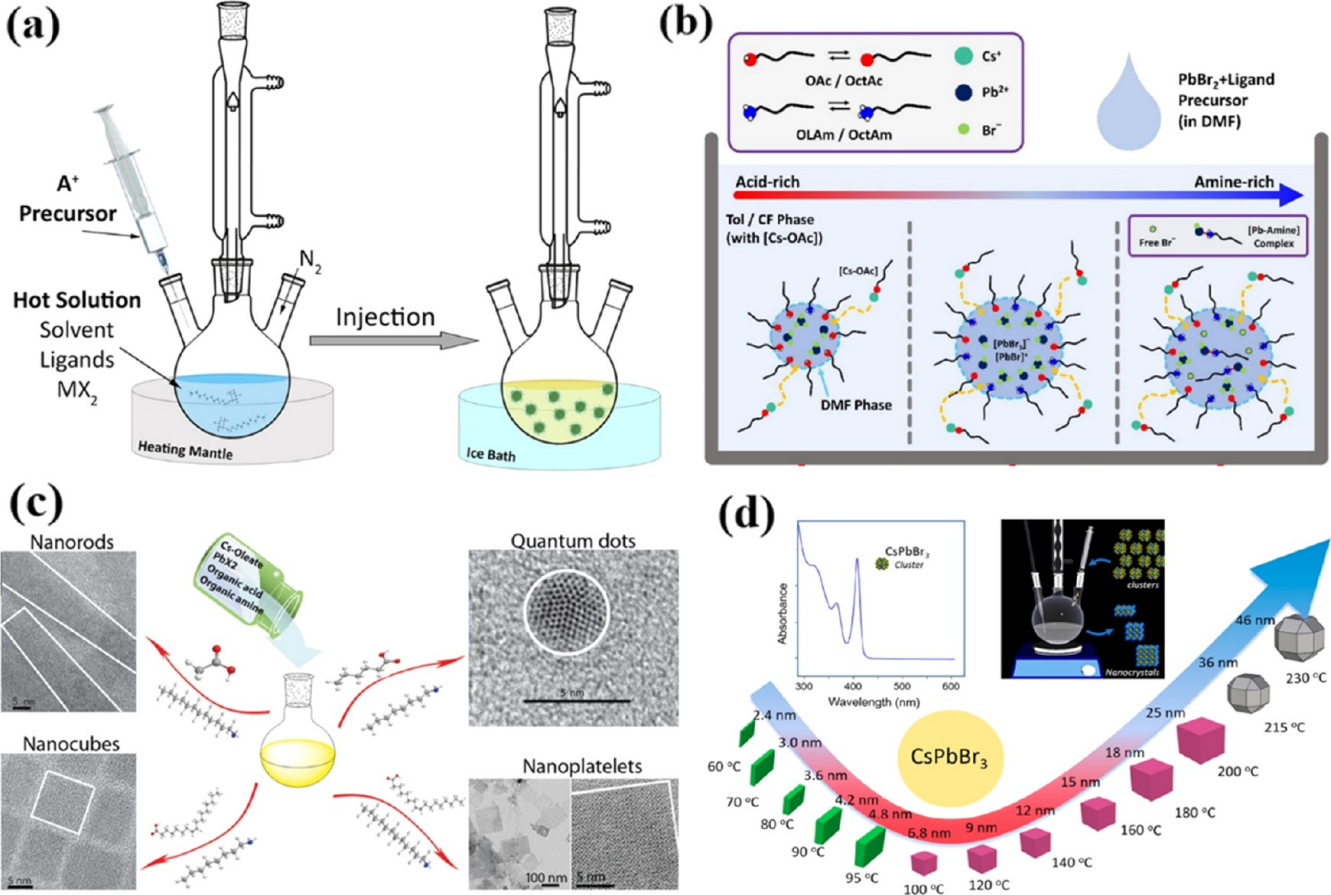
(a) Schematic diagram illustrating the hot-injection method employed in the synthesis of these nanocrystals.^32^ Reproduced with permission from ref (32). Copyright 2019 American Chemical Society. (b) Illustration outlining steps involved in the reaction procedure.^28^ Reproduced with permission from ref (28). Copyright 2023 ChemRxiv. (c) A schematic representation showcasing CsPbX_3_ nanocrystals with diverse morphologies achieved by modifying the organic acid and amine ligands under room temperature conditions.^29^ Reproduced with permission from ref (29). Copyright 2016 American Chemical Society. (d) The absorption spectrum of CsPbBr_3_ clusters at room temperature; the schematic representation of the injection process for cluster nanostructures into 1-octadecene to form nanocrystals; the schematic representation depicting the evolution of the shape and size of CsPbBr_3_ nanocrystals at different reaction temperatures.^33^ Reproduced with permission from ref (33). Copyright 2022 American Chemical Society.

As shown in Figure 2a, the first all-inorganic CsPbX_3_ nanocrystal was synthesized using the hot-injection method in 2015 by Protesescu et al.^27^ Nonetheless, the thermal-injection method necessitates elevated reaction temperatures and an inert gas environment to attain the high crystallinity of nanocrystals, consequently elevating the complexity and cost of experiments. As a result, researchers have begun to explore alternative approaches capable of synthesizing highly stable CsPbX_3_ nanocrystals under milder conditions, obviating the need for inert gas protection. Figure 2b illustrates the swift synthesis of CsPbX_3_ nanocrystals by using the ligand-assisted reprecipitation method at room temperature. In this process, a nonpolar solvent is rapidly introduced into a polar solvent containing Cs^+^, Pb^2+^, and X^–^. This results in the rapid nucleation and growth of gram-level CsPbX_3_ nanocrystals.^28^ Subsequently, numerous methods for synthesizing highly stable CsPbX_3_ nanocrystals have been swiftly developed. Irrespective of the chosen synthesis methods, it is essential to note that the shape of the resulting nanocrystals is primarily influenced by factors such as the specific ligands employed and the reaction temperature. As depicted in Figure 2c, the choice of specific ligands, such as hexanoic acid and octylamine, can result in the formation of spherical quantum dots. Conversely, the combination of oleic acid and dodecylamine leads to the creation of nanocubes. This illustrates how the selection of different ligand combinations can precisely tailor the shape and morphology of the nanocrystals in the synthesis process.^29^ Furthermore, previous research has revealed that lower reaction temperatures tend to promote the creation of quasi-2D nanoplatelets, while higher temperatures favor the generation of nanocubes. This temperature-dependent effect underscores the critical role of reaction conditions in controlling the shape and structure of the synthesized nanocrystals.^30^ Pan et al. have additionally noted that shape selectivity is influenced by the chain length of amines, with varying chain lengths leading to different nanocrystal shapes. However, they found that the choice of carboxylic acids has a comparatively lesser impact on shape selectivity. This insight further underscores the nuanced interplay of reaction components in nanocrystal synthesis.^31^

In Figure 2d (top left panel), a representative absorption spectrum of CsPbBr_3_ clusters is depicted. The pronounced peak at approximately 400 nm corresponds to the bandgap of the material, while the additional peaks at 353 and 318 nm are associated with higher quantum states within the spectrum. Additionally, microscopic imaging was conducted to visualize the assembly of these clusters, providing valuable insights into their structural organization and behavior. In Figure 2d (top right panel), a schematic representation is provided to illustrate the synthesis protocol utilizing clusters as the sole precursor to generate nanocrystals of varying sizes. Figure 2d (bottom panel) illustrates a variety of CsPbBr_3_ nanocrystals that can be achieved by injecting clusters at different temperatures. Below 100 °C, the typical outcome was the generation of thickness-tunable platelets. And in the temperature range from 100 to 200 °C, nanocrystals with tunable sizes in a cubic shape were produced. However, when the temperature was above 220 °C, the exclusive product formed was polyhedron-shaped rhombicuboctahedron nanocrystals.

**Figure 3:**
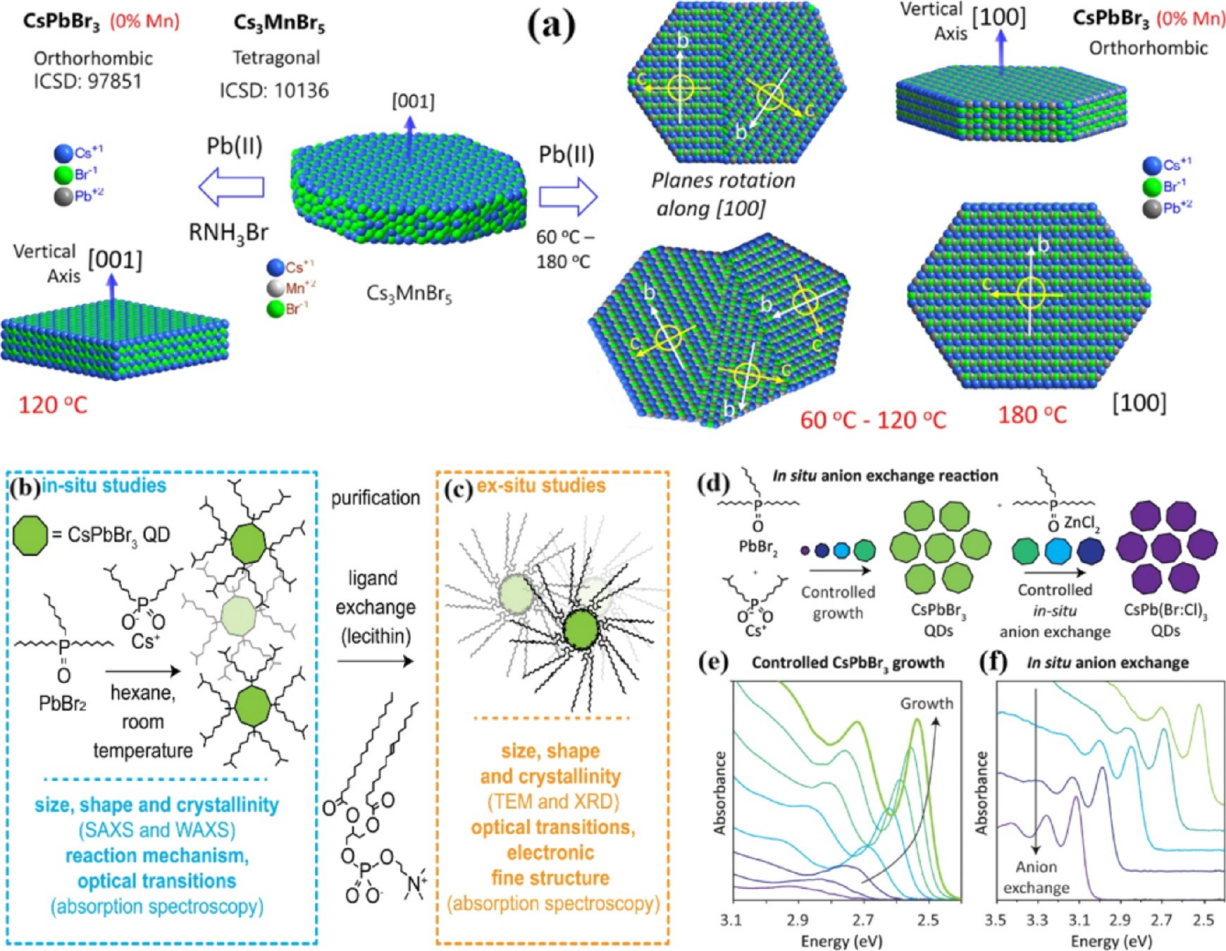
(a) Schematic presentation of the transformation of tetragonal Cs_3_MnBr_5_ to orthorhombic CsPbBr_3_ discs at different reaction temperatures.^34^ Reproduced with permission from ref (34). Copyright 2022 American Chemical Society. (b) The reaction scheme and an overview of *in situ* monitoring techniques are provided, allowing for real-time observation and analysis of the synthesis process.^35^ (c) Overview of used *ex situ* techniques on ligand-exchanged and washed quantum dots.^35^ Panels (b) and (c) are reproduced with permission from ref (35). Copyright 2022 Science. (d) The overall scheme depicts the combined slow growth process of CsPbBr_3_ quantum dots along with the *in situ* anion-exchange step, leading to the formation of CsPb(Cl:Br)_3_ quantum dots.^36^ (e) The gradual and controlled growth of 6.4 nm CsPbBr_3_ parent quantum dots, revealing multiple distinct excitonic absorption transitions.^36^ (f) The *in situ* anion exchange is executed immediately after quantum-dot growth, resulting in 6.4 nm CsPb(Br:Cl)_3_ quantum dots with varying Cl:Br ratios.^36^ Panels (d), (e), and (f) are reproduced with permission from ref (36). Copyright 2022 American Chemical Society.

Recently, Suman et al. successfully synthesized CsPbBr_3_ disk nanocrystals.^34^ As shown in Figure 3a, the tetragonal phase of Cs_3_MnBr_5_ was chosen as the parent material to serve as a Cs sublattice platform. Subsequently, these Cs_3_MnBr_5_ structures were introduced into a solution containing Pb(II) at various reaction temperatures to facilitate the formation of the desired lead-halide perovskite nanostructures. This process likely allowed for the controlled incorporation of Pb(II) into the Cs sublattice, leading to the creation of specific nanostructures with tailored properties. Interestingly, when the reaction temperature was lowered to 60 °C from the initial 120 °C, the resulting discs exhibited a different morphology. At the lower temperature, the number of junctions in the formed discs increased significantly, with an average of more than four junctions per disc. This temperature-dependent effect highlights the precise control over the structure of the synthesized nanostructures based on reaction conditions.^34^ These findings emphasize the significance of the temperature and reactant conditions in shaping the final nanostructure morphology.

As depicted in Figure 3b,c, Akkerman and colleagues devised a synthetic approach that activated the formation of PbBr^3–^ anions by exclusively introducing Cs cations as the sole available cation in the system throughout the entire synthesis process. This approach likely provided precise control over the reaction dynamics and ultimately influenced the properties of the resulting materials.^35^ Furthermore, Akkerman and colleagues achieved the synthesis of monodisperse CsPbBr_3_, CsPb(Cl:Br)_3_, and CsPbCl_3_ quantum dots (QDs) through a two-step process. Initially, they conducted a controlled, slow growth of CsPbBr_3_ QDs. Subsequently, they performed a controlled in situ anion exchange using ZnCl_2_, as demonstrated in Figure 3d. This approach allowed for the precise tailoring of QD compositions and properties through controlled growth and anion exchange steps.^36^ As depicted in Figure 3e,f, this controlled anion exchange facilitated the replacement of bromide ions (Br^–^) with chloride ions (Cl^–^) while maintaining the shape and excitonic absorption peaks of the QDs. Therefore, this method provided a means for meticulous control over the composition and properties of QDs while preserving their essential characteristics.

Different synthetic methods based on achieving different properties have been discussed above. However, the selection of ligands and dopants also plays a crucial role in achieving different properties. Ligands are organic molecules that bind to the surface of nanocrystals, affecting their growth, stability, and electronic properties. Common ligands used in the synthesis of perovskite NCs include oleic acid (OA), oleylamine (OLA), and octadecene (ODE). OA helps in stabilizing nanocrystals by preventing aggregation and controlling crystal growth, while the OLA acts similarly to OA but can also influence the electronic properties of the NCs. Normally, the ODE provides a medium for the reaction and assists in controlling the size and shape of the nanocrystals. Dopants in perovskite NCs can be generally categorized into two types based on the site they occupy within the crystal structure: A-site dopants and B-site dopants. A-site dopants include organic cations such as methylammonium (MA^+^) or formamidinium (FA^+^) and inorganic cations such as cesium (Cs^+^) or rubidium (Rb^+^). These cations typically influence the stability of the crystal structure and electronic properties. B-site dopants include transition-metal ions such as manganese (Mn^2+^), lead (Pb^2+^), or cadmium (Cd^2+^) and trivalent cation such as aluminum (Al^3+^) or bismuth (Bi^3+^). These cations are used to alter the electronic and optical properties of the nanocrystals, enhancing the photoluminescence and stability. Overall, ligands and dopants will be selected based on their ability to meet the specific requirements of the NCs, such as desired optical properties, stability against environmental factors, and compatibility with other components in devices. The summarized details of various synthetic methods for cation-doped cesium lead-halide perovskite nanocrystals (PeNCs) are presented in Table 1, offering a comprehensive overview of the different approaches used in their fabrication.

**Table 1:** Summary of Different Synthetic Methods for Cation-Doped Cesium Lead-Halide PeNCs

PeNCs composition	precursor	solvent	ligand	dopant type	synthetic method used	PLQY (%)	ref
CsPb_(0.94–0.97)_ Mn_(0.03–0.06)_Br_3_	Cs_2_CO_3_, PbBr_2_, MnI_2_	ODE, toluene	OA	bivalent (Mn^2+^)	postsynthetic cation, exchange (RT, photoinduced)	41–67	(37−39)
CsPb_0.6_Sn_0.4_I_3_	Cs_2_CO_3_, PbI_2_, SnI_2_,	ODE, toluene	OA	bivalent (Sn^2+^)	hot injection (170 °C, 5 s)	3	(40)
CsPb_0.77_Sn_0.23_Br_3_	Cs_2_CO_3_, PbBr_2_, SnBr_2_,	ODE, toluene	OA	bivalent (Sn^2+^)	postsynthetic cation exchange (RT, 16 h)	37–71	(37−48)
CsPb_0.95_Cd_0.05_ Br_3_	Cs_2_CO_3_, PbBr_2_, CdBr_2_·4H_2_O	ODE, toluene	OA	bivalent (Cd ^2+^)	postsynthetic cation exchange (RT, 16 h)	>60	(48)
CsPb_0.95_Cd_0.05_Cl_3_	Cs_2_CO_3_, PbCl_2_, Cd-acetate	ODE, toluene	OA	bivalent (Cd ^2+^)	hot injection (200 °C, 10 s)	8	(49)
CsPb_1–*x*_Cd*_x_*Cl_3_	Cs_2_CO_3_, Pb-acetate, benzoyl chloride, Cd-oleate	ODE, CHCl_3_	OA	bivalent (Cd ^2+^)	postsynthetic cation exchange (RT, 180 s, sonication assistance)	98	(50)
CsPb_0.74_Zn_0.26_I_3_	Cs_2_CO_3_, PbI_2_, ZnI_2_	ODE, toluene	OLA, OA	bivalent (Zn ^2+^)	hot injection (170 °C, 5 s)	98.5	(51)
CsPb_0.95_Zn_0.05_Br_3_	Cs_2_CO_3_, PbBr_2_, ZnBr_2_,	ODE, toluene	OLA, OA	bivalent (Zn ^2+^)	postsynthetic cation exchange (RT, 16 h)	>60	(48)
CsPb_0.93_Cu_0.07_Br_3_	Cs_2_CO_3_, PbBr_2_, CuBr_2_	ODE, toluene	OLA, OA	bivalent (Cu ^2+^)	hot injection (185 °C, several second)	95	(52)
CsPb_0.94_Ni_0.06_l_3_	Cs_2_CO_3_, PbI_2_, NiI_2_	ODE	OLA, OA	bivalent (Ni ^2+^)	hot injection (170 °C, several second)	81	(53)
CsPb_0.9_Ni_0.1_Cl_3_	Cs_2_CO_3_, PbCl_2_, NiCl_2_·*x*H_2_O	ODE	OLA, OA	bivalent (Ni ^2+^)	hot injection (210 °C, 60 s)	96.5	(54)
CsPb_0.97_Sr_0.03_l_3_	Cs_2_CO_3_, PbI_2_, SrI_2_	ODE	OLA, OA	bivalent (Sr ^2+^)	hot injection (170 °C, 5 s)	94	(55)
CsPb_1–*x*_Mg*_x_*Br_3_	Cs_2_CO_3_, PbBr_2_, MgBr_2_,	ODE	OLA, OA	bivalent (Mg^2+^)	postsynthetic cation	87–100	(56)
CsPb_1–*x*_Mg*_x_*Cl_3_	Cs_2_CO_3_, PbCl_2_, MgCl_2_,	ODE	OLA, OA	bivalent (Mg^2+^)	exchange (RT, 1 h)	-	(57)
CsPb_0.93_Ni_0.07_Br_3_	Cs_2_CO_3_, PbBr_2_, NiO	ODE	OA	bivalent (Ni ^2+^)	ground and heat treatment (350 °C, 3 h)	79	(58)
CsPb_1–*x*_Ce*_x_*Cl_3_	Cs_2_CO_3_, PbCl_2_, CeCl_3_·6H_2_O	ODE, toluene	OLA, OA	Trivalent (Ce^3+^)	hot injection (180 °C, 30 s)	4.8	(59)
CsPb_1–*x*_Sm*_x_*Cl_3_	Cs_2_CO_3_, PbCl_2_, SmCl_3_·6H_2_O	ODE, toluene	OLA, OA	trivalent (Sm^3+^)	hot injection (180 °C, 30 s)	4.9	(59)
CsPb_1–*x*_Eu*_x_*Cl_3_	Cs_2_CO_3_, PbCl_2_, EuCl_3_·6H_2_O	ODE, toluene	OLA, OA	trivalent (Eu^3+^)	hot injection (180 °C, 30 s)	5.4	(59)
CsPb_1–*x*_Tb*_x_*Cl_3_	Cs_2_CO_3_, PbCl_2_, TbCl_3_·6H_2_O	ODE, toluene	OLA, OA	trivalent (Tb^3+^)	hot injection (180 °C, 30 s)	5.2	(59)
CsPb_1–*x*_Dy*_x_*Cl_3_	Cs_2_CO_3_, PbCl_2_, DyCl_3_·6H_2_O	ODE, toluene	OLA, OA	trivalent (Dy^3+^)	hot injection (180 °C, 30 s)	6	(59)
CsPb_1–*x*_Er*_x_*Cl_3_	Cs_2_CO_3_, PbCl_2_, ErCl_3_·6H_2_O	ODE, toluene	OLA, OA	trivalent (Er^3+^)	hot injection (180 °C, 30 s)	6.1	(59)
CsPb_1–*x*_Yb*_x_*Cl_3_	Cs_2_CO_3_, PbCl_2_, YbCl_3_·6H_2_O	ODE, toluene	OLA, OA	trivalent (Yb^3+^)	hot injection (180 °C, 30 s)	6.4	(59)
abbreviation definition	octadecene (ODE); oleic acid (OA); oleylamine (OLA); room temperature (RT)						

## 3 Structural and Optoelectronic Properties of Perovskite Nanocrystals

### 3.1 Crystal and Electronic Structure

**Figure 4:**
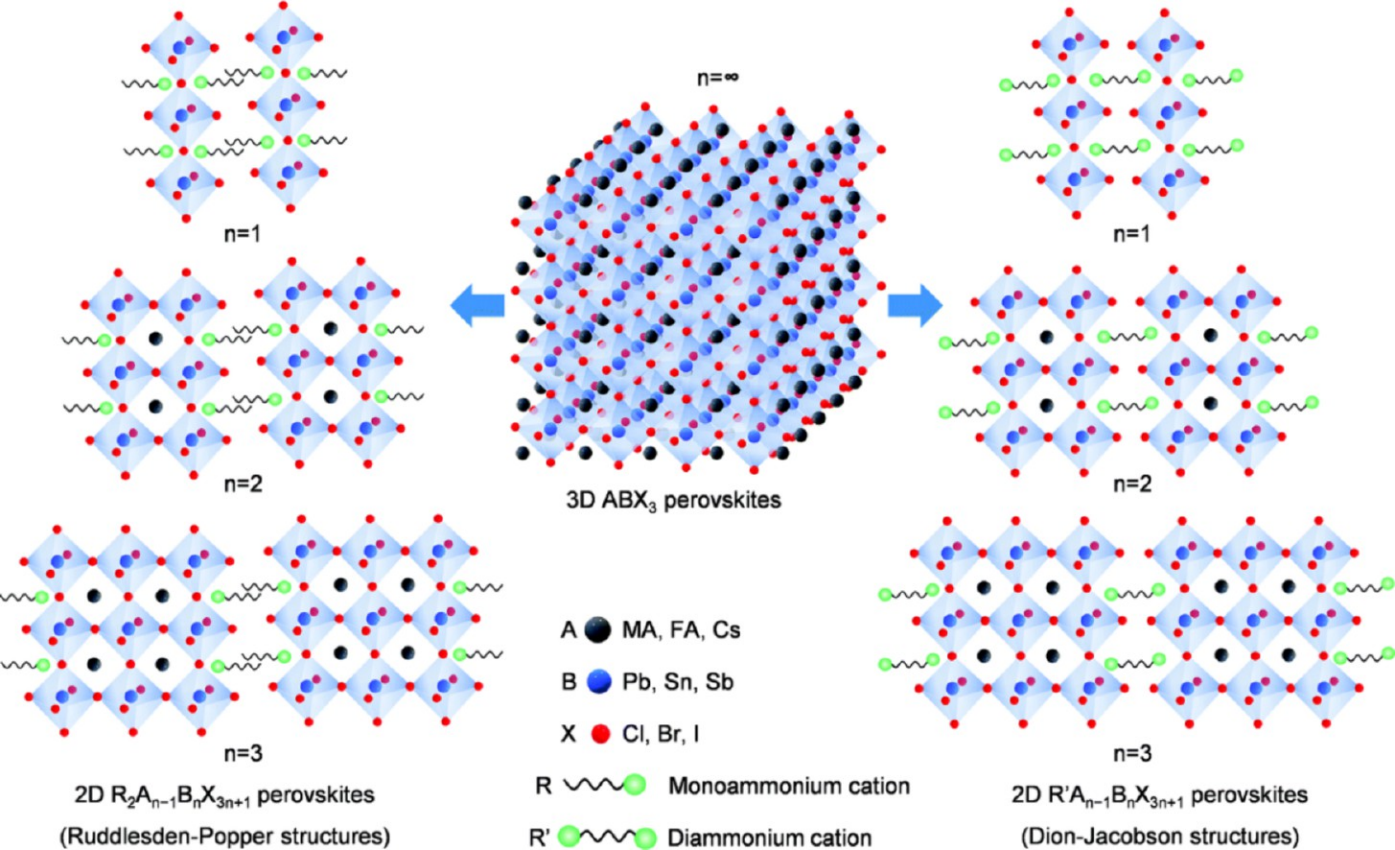
Crystal structures of 2D and 3D halide perovskites.^60^ Reproduced with permission from ref (60). Copyright 2021 Royal Society of Chemistry.

Numerous research studies on lead-halide perovskite nanocrystals have predominantly focused on NCs with a 3D APbX_3_ crystal structure and composition. However, the reactivity and intrinsic toxicity associated with this class of halide perovskites have spurred research efforts in various directions. The high ionicity and structural instability of LHP NCs, which can limit their applicability in certain contexts, have also been viewed as advantageous features. This is because the APbX_3_ lattice can be readily reorganized into different phases. Consequently, extensive investigations have been initiated into nanocrystals with alternative structures and compositions, often referred to as “perovskite-related structures”. These endeavors aim to expand the utility and versatility of halide perovskite nanocrystals in diverse applications. Figure 4 provides a clear distinction between the layered 2D and 3D halide perovskite structures.^60^ In the case of layered 2D halide perovskites, the flexibility, arrangement, and separation of inorganic stacking are influenced by the selection of organic spacer cations. Metal halide perovskites, known for their highly stable structures and exceptional optoelectronic and photophysical properties, have found extensive applications across various industries.

**Figure 5:**
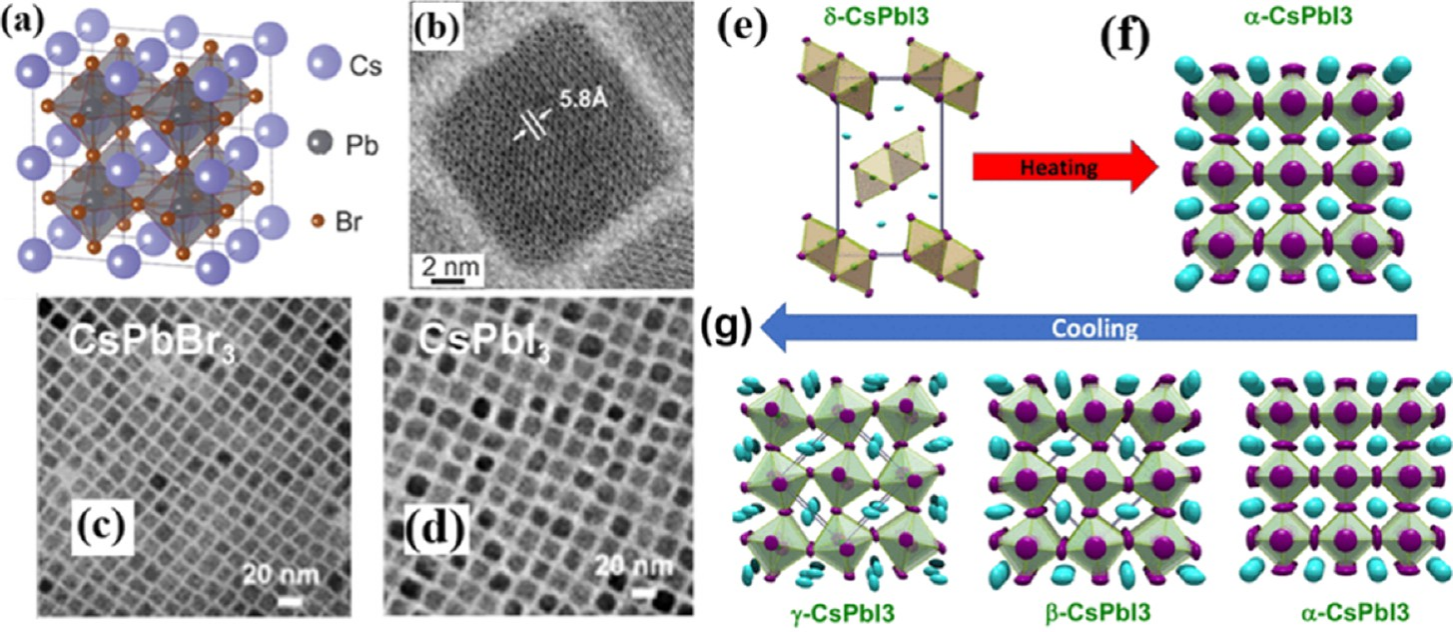
(a) Illustration of the cubic lattice structure in CsPbBr_3_ nanocrystals.^62^ (b) Transmission electron microscopy (TEM) micrograph of CsPbBr_3_ nanocrystals.^62^ Panels (a) and (b) are reproduced with permission from ref (62). Copyright 2017 Electrochemical Society; (c) TEM image of the CsPbBr_3_ thin film;^8^ (d) TEM image of CsPbI_3_ thin films.^8^ Panels (c) and (d) are reproduced with permission from ref (8). Copyright 2019 American Chemical Society; (e) δ phase CsPbI_3_; (f) α phase CsPbI_3_; and (g) phase transitions of CsPbI_3_.^61^ Panels (e), (f), and (g) are reproduced with permission from ref (61). Copyright 2018 American Chemical Society.

Varying the halide composition at the X-site in CsPbX_3_ nanocrystals allows for precise tuning of their bandgap, leading to different light-emission colors. For instance, CsPbI_3_ nanocrystals are capable of emitting red light, and they can adopt four distinct crystal structures: δ phase (nonperovskite structure); α phase (cubic); β phase (tetragonal); and γ phase (orthorhombic). These different crystal structures provide researchers with additional means to tailor the optical properties and characteristics of CsPbI_3_ nanocrystals for various applications.^61^ As reported by Marronnier et al., the structure of the CsPbBr_3_ perovskite nanocrystals is cubic, as depicted in Figure 5a. This cubic structure is a fundamental aspect of the crystal lattice for CsPbBr_3_, influencing its optical and electronic properties.^27^ Cubic crystals, such as those observed in CsPbBr_3_ perovskite nanocrystals, can sometimes exhibit parallel crystal edges that appear as stripes. These stripes result from the formation of twin crystals during the transformation from the high-temperature variant to the low-temperature variant. Twinning is a phenomenon where two or more crystals share a common boundary plane and can occur during crystal growth or phase transitions, leading to the observed parallel edge patterns in the cubic crystals.^27,61^ In the structure of the high-temperature variant of CsPbBr_3_, the lead ions are arranged in octahedral coordination, surrounded by six oxygen ions, resulting in a coordination number of six for lead. Meanwhile, cesium ions are positioned within a cavity formed by an octahedron, giving them a coordination number of 12.

Figure 5b illustrates that the size of CsPbBr_3_ perovskite nanocrystals is approximately 8 nm (nm), providing a visual representation of their dimensions at the nanoscale.^61^ The surface morphology and arrangement matrix of CsPbI_3_ and CsPbBr_3_ thin films are visible in Figure 5c,d, respectively. These images were captured using transmission electron microscopy (TEM), allowing for the detailed observation of the nanoscale features and structural characteristics of the thin films.^8^ The CsPbX_3_ cubic lattices exhibit uniform distribution, with the size of the CsPbBr_3_ cubic lattice measuring approximately 15 nm, slightly smaller than the CsPbI_3_ cubic lattice, which is around 20 nm in size. Figure 5e–g illustrates the connection between the temperature and structural phase transitions in CsPbI_3_. When the temperature exceeds the transition temperature, the initial δ phase in Figure 5e undergoes a transformation into the α phase in Figure 5f. Upon cooling, the α phase remains stable and can be supercooled below the transition temperature. As depicted in Figure 5g, if the temperature is maintained at room temperature, then the α phase will initially transition into the β phase and subsequently into the γ phase. It is important to note that the α, β, and γ phases are metastable and will eventually convert to the thermodynamically stable δ phase.

**Figure 6:**
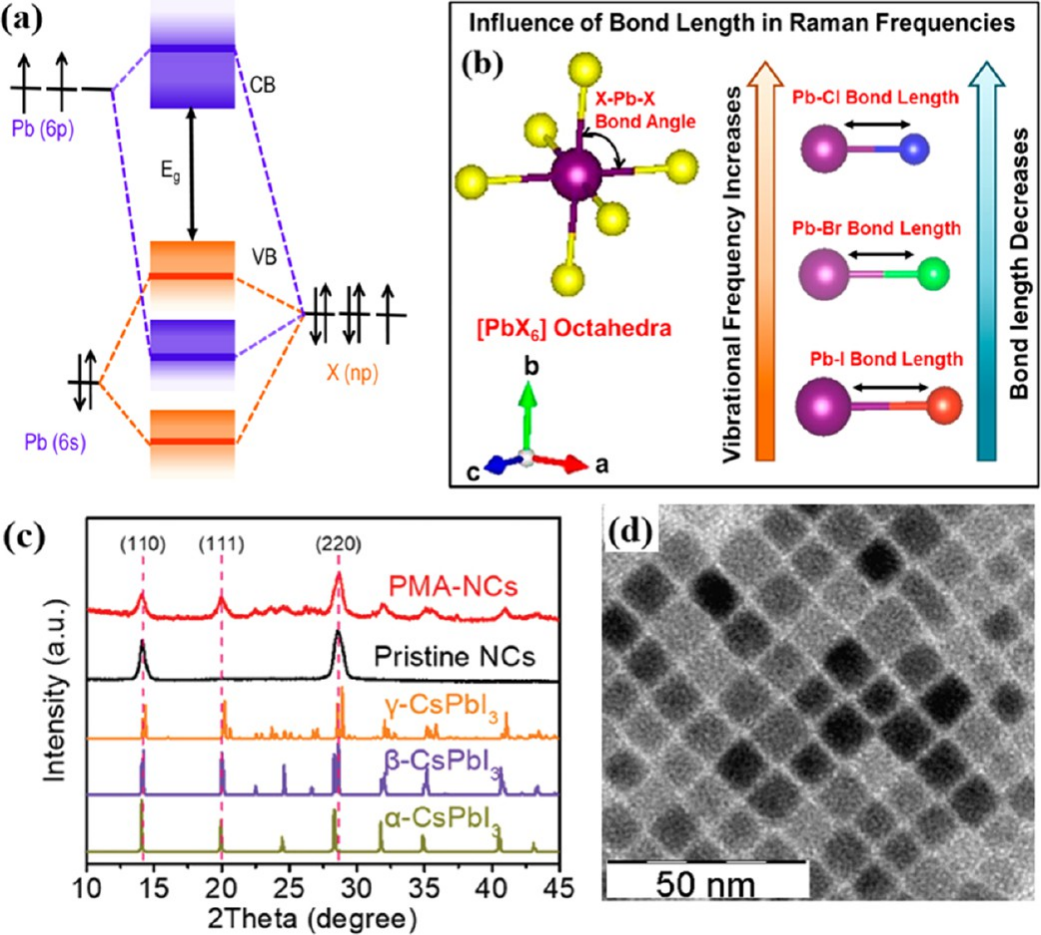
(a) APbX_3_ bonding/antibonding orbitals.^9^ Reproduced with permission from ref (9). Copyright 2016 American Chemical Society. (b) Electronic profiles.^64^ Reproduced with permission from ref (64). Copyright 2020 American Chemical Society. (c) X-ray diffraction (XRD) spectra of CsPbI_3_ nanocrystal films with varying phase structures.^61^ Reproduced with permission from ref (61). Copyright 2018 American Chemical Society. (d) Transmission electron microscopy (TEM) images of pristine CsPbBr_3_ NCs.^9^ Reproduced with permission from ref (9). Copyright 2016 American Chemical Society.

The APbX_3_ bonding/antibonding orbital diagram, as shown in Figure 6a, illustrates the creation of the conduction band and valence band maxima, with the bandgap lying between these two antibonds. In the case of CsPbX_3_, the valence band maximum (VBM) is primarily determined by the 6s orbital of Cs and the np orbitals of Pb, where the contribution from X (halogen) np orbitals is predominant.^9^ Consequently, as one progresses from iodide (with a 5p orbital) to bromine (with a 4p orbital) and further to chlorine (with a 3p orbital), the energy associated with the p orbital in the CsPbX_3_ halide system will decrease. As a consequence of this energy shift, the VBM will shift toward a higher, more positive potential. This change in energy levels affects the electronic structure and properties of the material, including its optical and electronic characteristics.^9^ According to density functional theory (DFT), the energy band structure of CsPbX_3_ perovskites is minimally affected by changes in the halide composition. This is primarily due to the presence of spin–orbit interactions and relativistic corrections.

Furthermore, all CsPbX_3_ perovskites exhibit a direct bandgap, which makes them promising candidates for applications in optoelectronic devices. While the electronic structure of A^+^ cations may be indirectly influenced by Cs^+^ cations through the distortion of the PbI_6_ lattice, it does not have a direct impact on the electronic structure of A^+^ cations at the band edge. Therefore, as depicted in Figure 6b, recombination and excitation of electrons occur exclusively within the PbX_6_ octahedron. This insight helps in understanding the behavior of charge carriers within the perovskite material.^64^ The geometric structures of CsPbBr_3_ and CsPbCl_3_ exhibit minimal changes when they transition between phases. In contrast, CsPbI_3_ is significantly affected by the ionic size of I^–^ and Pb^+^ ions, leading to substantial alterations in both its electronic and geometric properties, particularly in terms of the bond length and bandgap. This sensitivity to ionic size variations highlights the tunability of CsPbI_3_ and its potential for various applications where fine control over electronic properties is essential.^64^

Moreover, Li et al. confirmed that the structures of synthesized β-CsPbI_3_ NCs were crystallized by X-ray diffraction (XRD). Both pristine NCs and PMA-NCs display prominent peaks at 14.2 and 28.6° in the XRD spectra (Figure 6c).^61^ These peaks correspond to the (110) and (220) reflections of the tetragonal phase of CsPbI_3_. To gain a more in-depth understanding of the microstructure, the TEM image was employed to examine the detailed characteristics of these β-CsPbI_3_ nanocrystals (Figure 6d).^9^ The average particle size of the pristine NCs is 21.3 nm. High-resolution transmission electron microscopy (HRTEM) images reveal lattice fringes around 0.63 nm in the pristine NCs, which can be attributed to the (110) plane of β-CsPbI_3_. In the case of the PMA-NCs sample, the HRTEM images show lattice fringes of 0.63 and 0.44 nm, corresponding to the (110) and (111) planes of β-CsPbI_3_, respectively. These findings are in excellent agreement with the earlier XRD results and are consistent with what has been reported in the literature.^9^

### 3.2 Optoelectronic and Photophysical Properties

**Figure 7:**
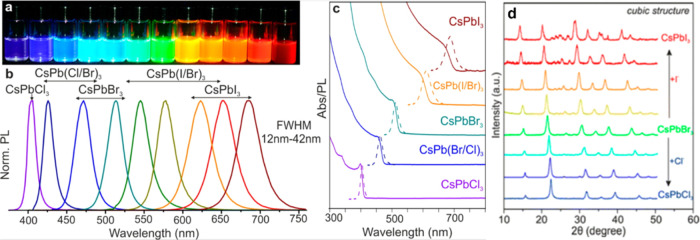
(a) CsPbX_3_ in toluene solution under UV light;^27^ (b) PL spectra;^27^ (c) UV–vis spectra;^27^ Panels (a), (b), and (c) are reproduced with permission from ref (27). Copyright 2015 American Chemical Society; and (d) XRD spectra of CsPbX_3_ nanocrystals.^11^ Reproduced with permission from ref (11). Copyright 2018 American Chemical Society.

CsPbX_3_ nanocrystals are known for their exceptional optoelectronic properties, making them promising candidates for use as luminescent materials in optoelectronic devices. They possess several advantageous characteristics, including the ability to achieve tunable electroluminescence, narrow emission bandwidth, high PLQY, and high EQE directly without the need for additional postprocessing steps. These attributes make CsPbX_3_ nanocrystals highly desirable for a wide range of optoelectronic applications.^3^ It is reliable that the photoluminescence emission of phosphide QDs and common metal chalcogenides can be highly sensitive to their granularity, which can result in low optical uniformity of the produced materials. Additionally, the emission color and photoluminescence peak position of CsPbX_3_ nanocrystals are determined by their halide composition. This sensitivity to both size and composition underscores the importance of precise control over these factors when working with these materials in optoelectronic applications.^11^ As demonstrated by Protesescu et al., as depicted in Figure 7a,b, the PL peak position of CsPbX_3_ nanocrystals can be adjusted to cover the entire visible color spectrum, ranging from 410 to 700 nm. This adjustability is achieved by fine-tuning the halide composition and ratio within the nanocrystals, allowing for precise control over the emission color and wavelength.^61^

Moreover, when iodine is utilized as the halide (X = I) in CsPbX_3_ nanocrystals, the photoluminescence peak position can extend to 700 nm, emitting a deep-red light. Conversely, when chlorine is the halide in CsPbX_3_ nanocrystals (X = Cl), the photoluminescence peak position decreases to 400 nm, resulting in deep-blue light emission. Similarly, when bromine is employed as the halide (X = Br) in CsPbX_3_ nanocrystals, the photoluminescence peak position is approximately 520 nm, leading to green light emission. As demonstrated in Figure 7c,d, cubic CsPbI_3_ nanocrystals exhibit the highest photoluminescence intensity among the CsPbX_3_ nanocrystals, while cubic CsPbCl_3_ nanocrystals display the lowest photoluminescence intensity. These findings emphasize the remarkable tunability of CsPbX_3_ nanocrystals, allowing for the fine control of the emission colors and intensities by manipulating their halide composition. This tunability is a valuable feature for tailoring these nanocrystals for various optoelectronic and photonic applications.^11,27^ The high resistance of CsPbX_3_ nanocrystals, which can withstand ultrahigh-density defects of up to 1–2 atomic percent, is an outstanding optoelectronic property. This resistance surpasses that of many other binary-composite QDs. It contributes to the achievement of higher PLQY, making CsPbX_3_ nanocrystals highly attractive for various optoelectronic applications where efficient light emission and performance stability are crucial.^11^ Furthermore, the surfaces of CsPbX_3_ nanocrystals can be passivated by using other semiconductors with wider bandgaps. This passivation strategy helps enhance the PLQY and stability of CsPbX_3_ nanocrystals, making them even more suitable for optoelectronic applications where improved performance and longevity are desired. Passivation techniques play a critical role in optimizing the properties of these nanocrystals for various practical uses.

Based on the discussion of the structures and properties of CsPbX_3_ nanocrystals, it is evident that high-performance LEDs may experience strong trap-assisted recombination. This phenomenon can be a primary factor contributing to the loss of photoluminescence efficiency in such devices.^18^ Reducing trap-assisted recombination is a key challenge in decreasing nonradiative recombination and enhancing the PLQY in high-performance LEDs based on halide perovskite nanocrystals. A highly effective approach to address this issue is A/B-site doping, which widens the bandgap of the halide perovskite material. Therefore, introducing dopants at the A and B sites of the perovskite lattice is a viable strategy for reducing the trap density within the material. This reduction in trap density helps mitigate trap-assisted recombination, ultimately resulting in improved PLQY. Additionally, the widening of the bandgap through doping enhances the optoelectronic properties of the material, making it a highly promising approach for optimizing the performance of high-performance LEDs and various other optoelectronic devices.

In recent years, CsPbX_3_ PeNCs have gained significant popularity as luminescent materials, primarily due to their exceptionally high PLQY. However, it is worth noting that the quantum yields of PeNCs can vary significantly depending on the specific types of halogens used. For instance, when prepared through hot-injection methods, red-emitting CsPbI_3_ PeNCs have achieved near-unity PLQY, indicating extremely efficient light emission,^20,66^ and the green-emitting CsPbBr_3_ PeNCs typically exhibit in the range of 60–80%.^67^ In contrast to red- and green-emitting CsPbX_3_ perovskite nanocrystals, the synthesis of blue-emitting CsPbCl_3_ has proven to be more challenging. Typically, the PLQY of CsPbCl_3_ nanocrystals that have not been doped or passivated is relatively low, often less than 10%. Achieving high-efficiency blue emission in CsPbCl_3_ nanocrystals has been a more complex task, and researchers have been working on various strategies to improve their PLQY for blue light applications.^68−73^ To achieve CsPbX_3_ PeNCs with high PLQY, exceptional stability, narrow emission line width, and bandgap modification, researchers have explored numerous optimization methods, including cation doping, anion doping, and surface passivation. These optimization strategies are essential for tailoring CsPbX_3_ PeNCs for specific applications in optoelectronics and photonics.^74^

The selection of solvents and precursors plays a crucial role in the synthesis of CsPbX_3_ PeNCs. Researchers have extensively studied and optimized these factors to achieve the desired properties and high PLQY in the synthesized PeNCs. Therefore, the selection of solvents and precursors is of paramount importance, as it profoundly influences the entire process of crystal growth, size distribution, and surface passivation during the synthesis of PeNCs. These factors are pivotal in achieving PeNCs with the specific optical and electronic properties desired for various applications. Researchers carefully consider and optimize these choices to tailor the PeNCs to their intended uses in optoelectronics and photonics. In this context, the primary focus is on summarizing the enhancement of PLQY achieved through doping at the B-site. This approach involves introducing divalent cations with the same valence as Pb^2+^ as well as some trivalent and tetravalent cations. Such doping strategies have proven to be effective in significantly improving the PLQY of PeNCs, which is crucial for optimizing their performance in various optoelectronic and photonic applications. Table 2 likely provides specific examples and data regarding the impact of these dopants on the PLQY.

**Table 2:** Summary of the PLQY Improvement of CsPbX_3_ NCs Brought by Doping with Different Cations

		Emission wavelength (nm)		PLQY (%)		
Host sample	Dopant used	Undoped	Doped	Undoped	Doped	Ref
CsPbCl_3_	Cd^2+^	406	406	3	96	(50)
CsPbCl_3_	Mg^2+^	403	403	1	79	(56)
CsPbCl_3_	Cu^2+^	415	406	7	22	(52)
CsPb(Cl/Br)_3_	Cu^2+^	466	453	23	80	(52)
CsPb(Cl/Br)_3_	Cu^2+^	466	488	23	78	(52)
CsPbBr_3_	Mg^2+^	516	513	51	100	(56)
CsPbBr_3_	Ce^3+^	516	510	41	89	(75)
CsPbBr_3_	Sn^4+^	510	510	45	83	(76)
CsPbBr_3_	Sb^3+^	460	460	50	74	(77)
CsPbBr_3_	Na^+^	530	509	44	85	(78)
CsPbBr_3_	Cd^2+^	510	510	51	98	(50)
CsPbBr_3_	Cu^2+^	517	506	85	95	(52)
CsPbBr_3_	Mn^2+^	515	515	53	57	(37)
CsPbI_3_	Mn^2+^	694	694	65	90	(37)

### 3.3 Approaches to Enhance the Stability of Halide Perovskite NCs

Over the past few years, extensive research and ongoing development efforts have led to halide perovskite LEDs achieving remarkable external quantum efficiency (EQE) exceeding 30%. However, a significant challenge persists due to the inherent susceptibility of the halide perovskite crystal structure to degradation in the presence of air and humidity. This instability remains the primary barrier to achieving commercial viability of these solar cells. To delve into the fundamental causes of this crystal phase instability, the Goldschmidt tolerance factor (*t*) serves as a reliable empirical indicator.^79^ The Goldschmidt tolerance factor can be determined using the following equation:
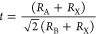
1

When the calculated tolerance factor falls within the range of 0.8 to 0.9, it indicates that the perovskite structure undergoes distortion, resulting in the formation of an oblique octahedron. Conversely, if the tolerance factor exceeds 1.11 or falls below 0.81, it signifies the formation of a nonperovskite structure.^80^ Perovskite materials exhibit an ideal cubic structure when the tolerance factor falls within the range of 0.9 to 1.0.^80^ In particular, when the tolerance factor approaches 1, the crystal structure of perovskite closely approximates a perfect 3D-cubic arrangement.^81−86^ In the case of the CsPbX_3_ perovskite, using CsPbI_3_ as an example, the small size of the Cs^+^ cation (181 pm) results in a tolerance factor for CsPbI_3_ that is close to the critical value of 0.8. This proximity to 0.8 leads to phase instability. Typically, CsPbI_3_ nanocrystals can exist in cubic (α), tetragonal (β), and orthorhombic (γ) phases, all of which exhibit active optoelectronic properties. This combination of phases is commonly referred to as the “black phase”.^61^

Additionally, there exists an orthogonal nonperovskite δ phase, which lacks active optoelectronic properties and is commonly known as the “yellow phase”.^87^ When the α-CsPbI_3_ sample is exposed to ambient air, the initially black α phase gradually transitions to a yellow phase. This phase change primarily occurs due to a reduction in the formation energy induced by moisture. Fafarman et al. were the first to suggest a solution involving doping to stabilize the crystal phase. They achieved this by introducing chloride ions at the X-site, effectively increasing the tolerance factor of CsPbI_3_. This chloride ion doping mechanism effectively inhibits the phase transition triggered by humidity.^88^ Following the successful anion doping method, further advancements were made by extending the approach to involve cation doping of both the A and B sites within the perovskite structure.

Doping plays a crucial role in enhancing the stability of the crystalline phases by increasing their formation energy. Consequently, there is extensive research focused on investigating various types and ratios of doping to improve the formation energy of inorganic halide perovskite crystalline phases.^55^ Yao et al. conducted research in which they controlled the size of the CsPbI_3_ quantum dots and introduced an innovative Sr^2+^ doping technique. Their findings demonstrated that Sr^2+^ doping plays a pivotal role in elevating the formation energy of α-CsPbI_3_, minimizing structural distortions, and enhancing the stability of the nanoscale cubic phase.^55^ Subsequently, they replaced the initially doped Sr^2+^ with Zn^2+^ and confirmed that this substitution also contributed to the increased stability of the crystal phase. As a result, the CsPbI_3_ nanocrystals doped with Zn could retain their cubic α-phase for a remarkable duration of 70 days when exposed to ambient air. Moreover, they achieved a near-unity PLQY with these Zn-doped CsPbI_3_ nanocrystals.^51^

In addition to the Goldschmidt tolerance factor, the octahedral factor (μ) is another parameter commonly employed to assess and express the stability of perovskite structures. These factors provide valuable insights into the structural characteristics and stability of perovskite materials,
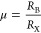
2

Certainly, the octahedral factor (μ) is defined as the ratio of the ionic radii of the species located at the B-site to those at the X-site within the perovskite crystal structure. This parameter helps evaluate the compatibility of the ions occupying these different positions, which in turn influences the stability and properties of the perovskite material.^66,67,69,89^ Within the domain of lead-halide perovskites, the octahedral factor (μ) required for the formation of a 3D-cubic perovskite nanocrystal typically falls within the range of 0.44 < μ < 0.90. This range of μ values is critical for maintaining the desired structural characteristics and stability of cubic perovskite nanocrystals.^70^ Maintaining the tolerance factor within the range of 0.81–1.11 and the octahedral factor in the range of 0.44–0.90 is a necessary condition for the formation of stable 3D-cubic perovskite nanocrystals. However, it is essential to note that while these factors are necessary, they are not always sufficient on their own. Other factors, such as the choice of dopants, synthesis methods, and environmental conditions, can also influence the stability and properties of perovskite nanocrystals. Therefore, a comprehensive approach that considers these factors in conjunction with appropriate μ and *t* values is typically required to ensure the successful formation and stability of 3D-cubic perovskite nanocrystals.^71^

There are many articles reporting on the different theoretical mechanisms by which doping can improve the stability of perovskite NCs. Jun showed that Bi^3+^ dopants introduce deep trap states that lead to PL quenching.^90^ The Ce^3+^ dopant enhances the CsPbBr_3_ lattice order and enriches the conduction band edge states through antisite CePb, resulting in PL enhancement.^90^ According to Dengfeng et al., AE^2+^ dopants can promote radiative recombination of carriers and promote intraband coupling by intrinsically reducing carriers trapped in intra- and inter-band defect states.^91^ Furthermore, the elimination of Br and Pb vacancies can enhance the short-range ordering of the CsPbBr lattice and enrich the conduction band edge states, resulting in enhanced PL of CsPbBr nanocrystals.^91^ Raihana et al., found that doping increases the energy difference between the states of the acceptor and donor parts of the molecule, thereby promoting the interfacial charge transfer process.^92^ Shenghan et al., confirmed that the significant improvement in thermal stability and optical properties of CsPbX_3_:Mn^2+^ QDs is mainly due to the successful doping of Mn^2+^ in CsPbX_3_ QDs, thereby increasing the formation energy.^37^ Besides, research by Yanan et al. shows that alkali metal cation-doped perovskite halides promote lattice shrinkage, crystallization kinetics, and electrical energy distribution.^94^ Moreover, Jia-Kai Chen et al., proposed a model for the observed anomalous incorporation of AE ions in NCs and achieved a PLQY of 77.1% for violet emission by incorporating an optimal amount of Ca.^94^ Furthermore, according to Sen’s research, the passivation effect of Na^+^ doping also greatly reduces nonradiative trap centers in NCs. In summary, the stability of perovskites can be greatly improved through different types and mechanisms of doping.

CsPbX_3_ nanocrystals possess outstanding optical properties, but they are highly sensitive to environmental factors such as oxygen, water, heat, and light. This sensitivity can lead to structural instability and decomposition. To address these challenges and stabilize the nanocrystals, ligands like oleic acid and oleylamine (OAm) are commonly used during the synthesis process. These ligands play a crucial role in passivating the surface of the nanocrystals, preventing surface defects, and enhancing their stability in ambient conditions. Proper ligand selection and passivation are essential to ensure the longevity and performance of CsPbX_3_ nanocrystals in practical applications. In the final purification and separation process, the removal of ligands from the surface of CsPbX_3_ nanocrystals can result in their collapse and aggregation, which may compromise their structural integrity and performance.^20^ To address this issue and improve the stability of CsPbX_3_ nanocrystals, new ligands or passivators are often introduced. These passivators can be categorized into four main groups: Alkali metal ions (K^+^, Rb^+^), organic cations (MA^+^, FA^+^), halogen anions (CI^–^, Br^–^, I^–^), and Lewis characteristic additives.^3^ Moreover, silicon oxide (SiO_*x*_) and aluminum oxide (AlO_*x*_) are additional passivation materials that can be used to effectively passivate the surface of CsPbX_3_ nanocrystals. These oxide-based passivators can enhance the stability of the nanocrystals and mitigate surface defects, improving their overall performance.^20^

**Figure 8:**
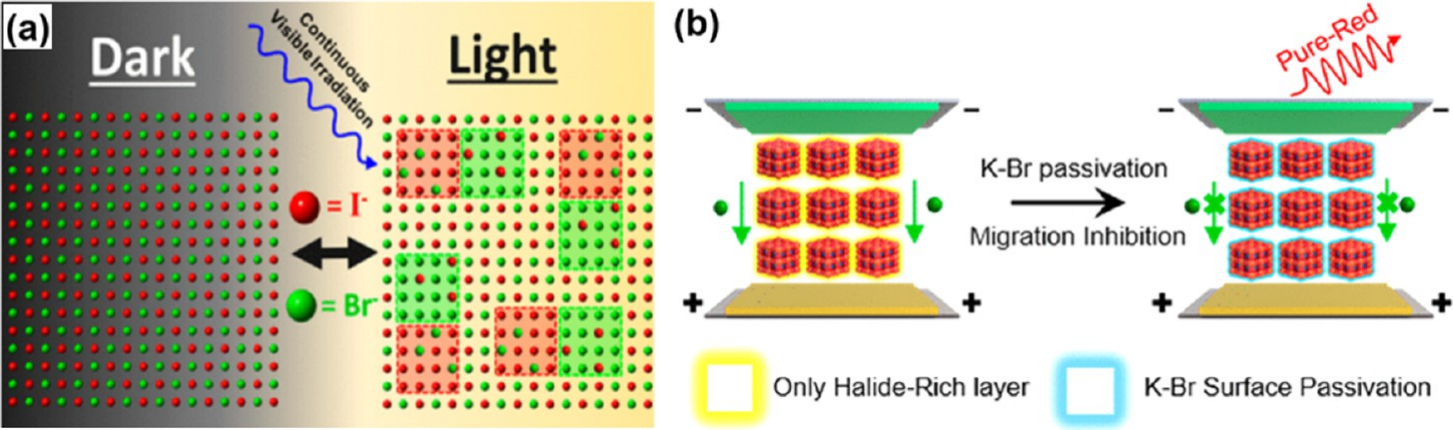
(a) Schematic of halogen ion migration and clusters under light.^96^ Reproduced with permission from ref (96). Copyright 2018 American Chemical Society. (b) Mechanism of surface passivation.^95^ Reproduced with permission from ref (95). Copyright 2020 American Chemical Society.

In Yang’s experiment, potassium oleate is employed as a passivating agent to coat and stabilize the surface of CsPbI_*x*_Br_3–*x*_ nanocrystals.^95^ As shown in Figure 8a, in perovskite AB(I_1–*x*_Br_*x*_)_3_ nanocrystals, particularly when *x* exceeds 0.2, there is a tendency for halide ions to segregate and form two distinct enrichment domains when exposed to light.^96^ In CsPbI_*x*_Br_3–*x*_ nanocrystals, the separation into bromide-rich and iodide-rich regions leads to varying bandgap energies. Bromide-rich areas have a higher bandgap, while iodide-rich regions have a lower bandgap. This can create low-energy phases, causing surface defects that reduce stability and PLQY. These issues directly impact their use in LEDs and similar devices. Strategies such as passivation can help mitigate surface defects and enhance nanocrystal performance. In Figure 8b, potassium and bromine ions form compounds that can treat surface defects in CsPbI_*x*_Br_3–*x*_ nanocrystals. This treatment improves nanocrystal performance by reducing defects, enhancing stability, and potentially increasing photoluminescence efficiency.^95^ As a result, the halide ions become immobilized and their movement is restricted. This advancement has led to cutting-edge green PeLEDs with a typical EQE of 25.2% and a peak EQE of 28.1%. These devices also exhibit an operating lifetime (T50) of 4.04 h when exposed to air without encapsulation, marking a significant improvement compared to undoped PeLEDs.^97^

## 4 Bandgap Tuning by A-Site Doping

### 4.1 Impact of A-Site Cation Doping on Optoelectronic Properties of Perovskite NCs

**Figure 9:**
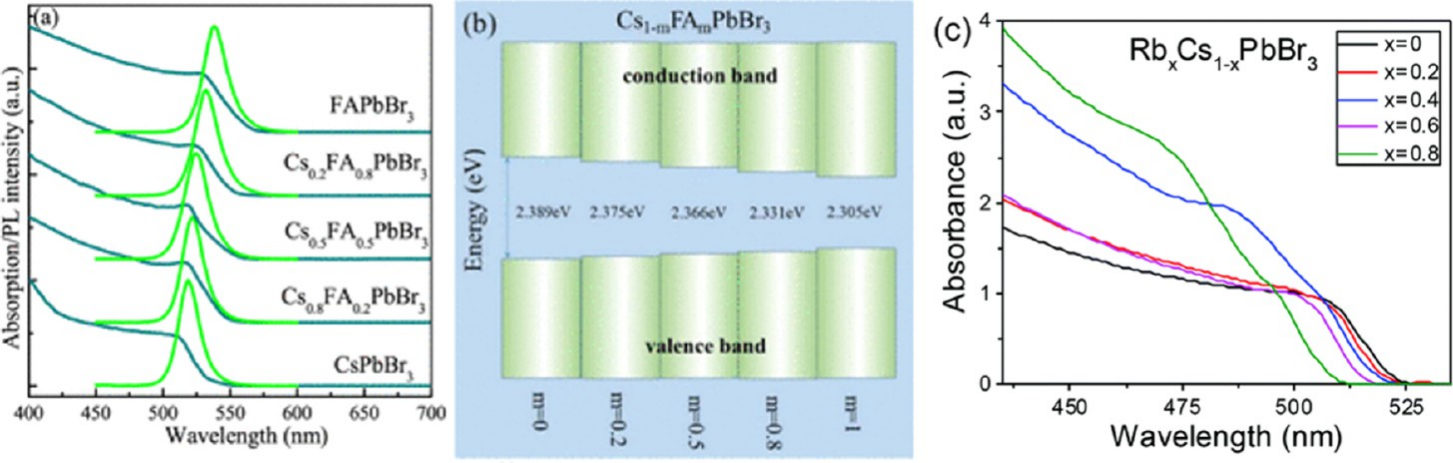
(a) Representative optical absorption/PL spectra (λ_ex_ = 375 nm).^73^ (b) Schematic illustration of a variation of the bandgap with an increase in the FA^+^ content in halide perovskite quantum dots (HPQDs).^73^ Panels (a) and (b) reproduced with permission from ref (73). Copyright 2017 American Chemical Society. (c) Absorbance of Rb_*x*_Cs_1–*x*_PbBr_3_ NPs (*x* = 0, 0.2, 0.4, 0.6, 0.8).^98^ Reproduced with permission from ref (98). Copyright 2018 Royal Society of Chemistry.

A-site dopants in perovskite nanocrystals play a critical role in modifying the optical properties of these materials. The A-site in perovskite structures typically hosts large cations and is crucial for stabilizing the crystal structure. Doping at the A-site can slightly increase or decrease the bandgap depending on the dopant size. Importantly, the initial phase structure of CsPbX_3_ nanocrystals remains unaffected on partially replacing Cs^+^ with other doping cations. This aspect holds significance for nanomaterial applications.^72^ In the case of CsPbX_3_, doping with larger ionic radius cations such as Formamidinium or Methylammonium leads to an increase in the bandgap. Chen et al. demonstrated that the bandgap of Cs_*x*_FA_1–*x*_PbBr_3_ QDs gradually increases with increasing FA ratio, which is shown in the UV spectrum (Figure 9a).^73^ Due to the doping of FA^+^, which has a large ionic radius, the bandgap of the perovskite is increased, resulting in the change of Pb–Br’s bond length and the angle of [PbX6]^4–^. Similar phenomena are also found in the Cs_*x*_MA_1–*x*_PbBr_3_ and Cs_*x*_FA_1–*x*_PbI_3_ systems.

On the other hand, as shown in Figure 9b, the bandgap will be decreased by incorporating smaller dopants.^73^ When smaller cations (such as Rb or K) are introduced, the opposite trend is observed for the incorporation of FA/MA. For example, Amgar et al.^98^ synthesized Rb_*x*_Cs_1–*x*_PbX_3_ quantum dots whose absorption band edges blue-shifted with an increasing Rb ratio (Figure 9c). Similarly, doping K to CsPbX_3_ nanocrystals has also resulted in a blue shift.^99,100^ Therefore, the bandgap of CsPbX_3_ perovskite is adjustable within a small range (no more than 0.1 eV) by A-site doping, resulting in changing the bond length of Pb–X and the angle of [PbX_6_] ^4–^.

As discussed in Section 3.3, the concept of the Goldschmidt tolerance factor (*t*) is often used to predict the stability of perovskite structures.^79,80^ Doping the A-site with different cations affects *R*_A_, thereby altering the Goldschmidt tolerance factor. If the factor is close to one, then the perovskite structure is ideally cubic, which suggests minimal distortion in the octahedra. Significant deviations from this ideal value lead to distortions in the bond angles and lengths as the structure adjusts to maintain stability. In terms of the changes in bond lengths and angles, there is a regular trend where smaller or larger A-site cations than the original can compress or expand the lattice, respectively.^80^ This compression or expansion directly influences the bond lengths and angles. For example, a smaller cation might lead to a compressed octahedral cage, decreasing the bond length and potentially altering the angle to accommodate the new lattice dimensions.^84,85^ However, the specific effects on bond lengths and angles can vary depending on the particular combination of the A-site cation and the original lattice configuration. However, the general principle that the lattice adjusts to accommodate the size and charge of the new A-site cation holds universally across different perovskite materials.^86^ In conclusion, while the specific magnitude and impact of changes in bond lengths and angles due to A-site doping can vary, the occurrence of these changes follows a regular and universal pattern governed by crystallographic and chemical principles. Various A-site dopants and their impacts have been shown in Table 3.^74^

**Table 3:** Summary of the Emission Peak Wavelength, Full Width at Half-Maximum (FWHM), and PLQY of Metal Halide Perovskite NCs with Different A-Site Dopants^74^

nanocrystal composition	emission peak (nm)	FWHM (nm)	PLQY (%)
CsPbCl_3_	390	∼25	—
MAPbCl_3_	407	∼25	—
FAPbCl_3_	413	∼25	—
CsPbBr_3_	510	∼15	—
MAPbBr_3_	532	∼15	—
FAPbBr_3_	541	∼15	—
CsPbI_3_	660	∼15	—
MAPbI_3_	756	∼15	—
FAPbI_3_	805	∼15	—
MA_0.9_Cs_0.1_PbBr_3_	539	∼17	—
MA_0.6_Cs_0.4_PbBr_3_	533	∼17	—
MA_0.9_Cs_0.1_PbI_3_	671	∼70	58
MA_0.8_Cs_0.2_PbI_3_	738	∼87	44
MA_0.7_Cs_0.3_PbI_3_	744	∼56	35
MA_0.5_Cs_0.5_PbI_3_	744	∼49	26
FA_0.9_Cs_0.1_PbBr_3_	∼531	∼20	∼73
FA_0.8_Cs_0.2_PbBr_3_	∼529	∼20	∼65
FA_0.7_Cs_0.3_PbBr_3_	∼525	∼20	∼54
FA_0.6_Cs_0.4_PbBr_3_	∼525	∼20	∼55
FA_0.5_Cs_0.5_PbBr_3_	∼520	∼20	∼47
FA_0.4_Cs_0.6_PbBr_3_	∼520	∼20	∼34
K^+^:CsPbCl_3_	∼405	∼15	2.08
K^+^:CsPbBr_3_	∼500	∼30	71.51
K^+^:CsPbI_3_	∼675	∼20	79.51
Rb_0.2_Cs_0.8_PbCl_3_	∼414	∼12	∼3
Rb_0.4_Cs_0.6_PbCl_3_	∼400	∼13	∼2
Rb_0.6_Cs_0.4_PbCl_3_	∼394	∼13	∼7
Rb_0.8_Cs_0.2_PbCl_3_			∼9
Rb_0.2_Cs_0.8_PbBr_3_	∼514	∼18	∼35
Rb_0.4_Cs_0.6_PbBr_3_	∼512	∼22	∼59
Rb_0.6_Cs_0.4_PbBr_3_	∼505	∼22	∼48
Rb_0.8_Cs_0.2_PbBr_3_	∼495	∼24	∼36
Rb_*x*_Cs_1–*x*_PbBr_3_	460–500	<25	60–90
Tl_3_PbI_5_	∼530	∼115	—

Except for the bandgap tuning, A-site doping also can modify the optical absorption and emission. Because the changes in the bandgap directly affect the optical absorption and emission properties of perovskites. A wider bandgap resulting from larger A-site dopants can shift the absorption edge toward the blue region of the spectrum, leading to emission of bluer light. Conversely, a smaller bandgap can shift the emission toward longer wavelengths, producing red or near-infrared light. This is crucial for applications like solar cells and LEDs where specific bandgap energies are needed for efficient operation. Moreover, A-site doping can also enhance the photoluminescence efficiency of perovskite nanocrystals. This improvement often results from the reduced nonradiative recombination pathways within the crystal. Certain dopants can help passivate defects within the crystal lattice, which are sites for nonradiative recombination. By the reduction in these defects, the dopants increase the likelihood of radiative recombination, thereby enhancing the photoluminescence efficiency. Besides, the choice of A-site cation can also influence the stability and phase purity of the perovskite nanocrystals. Dopants that better stabilize the perovskite crystal structure can lead to improved material durability and less degradation under operating conditions, which are beneficial for the longevity and performance consistency of photovoltaic cells and LEDs. Furthermore, A-site dopants can affect how charge carriers (electrons and holes) move through the perovskite material. By altering the lattice dimensions and symmetry through doping, the mobility of charge carriers can be optimized, which directly influences the overall electronic and optoelectronic properties of the material. In summary, A-site doping is a powerful tool for engineering the optical and electronic properties of perovskite nanocrystals. By carefully selecting appropriate A-site dopants, researchers can tailor these materials for specific applications, optimizing their performance in LED devices.

### 4.2 Impact of A-Site Doping on Perovskite LED Performance

**Figure 10:**
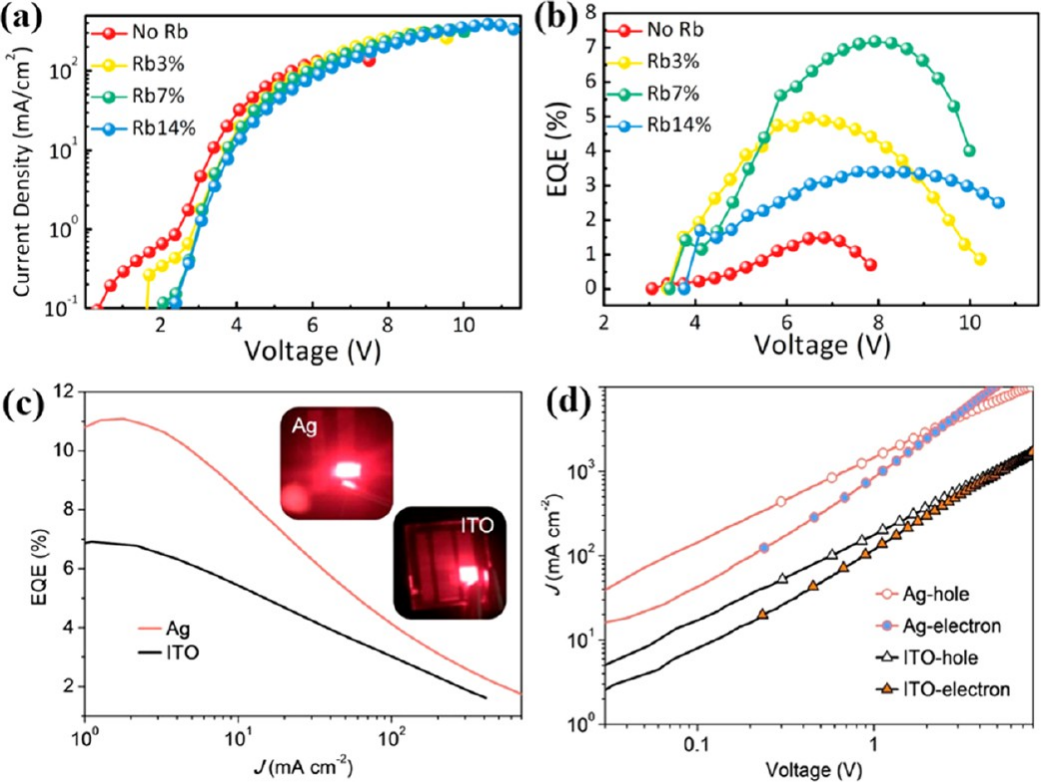
(a) Current density–voltage and (b) EQE–voltage curves of PeLEDs based on the FAPbBr_3_ film incorporated by different Rb contents.^101^ Panels (a) and (b) are reproduced with permission from ref (101). Copyright 2018 American Chemical Society. (c) EQE current density of CsPbI_3_ and Ag-doped CsPbBr_3_ LEDs;^106^ (d) current density as a function of voltage curves of Ag/ITO electron/hole LEDs.^106^ Panels (c) and (d) are reproduced with permission from ref (106). Copyright 2018 American Chemical Society.

The performance of LED can be improved by A-sites doping with two or three mixed cations.^101−103^ According to Shi et al.,^101^ as shown in Figure 10a, when Rb ions are doped into the perovskite emissive layer, the breakdown voltage of the PeLEDs shows an increasing trend (from 9.6 to 11 V) as the increasing concentration of Rb, which indicates that Rb doping can enhance the durability of PeLEDs device. Moreover, as the concentration of Rb increases from 0 to 7%, the EQE also increases significantly, as shown in Figure 10b. Song et al, also reported that the exciton trap can be decreased and the recombination efficiency of CsPbBr_3_ NCs can be improved by FA^+^ doping, resulting in a slight reduction in current, indicating that FA^+^ has suppressed the charge imbalance.^104^ Ultimately, compared with pure CsPbBr_3_, the brightness of the FA-doped CsPbBr_3_ LEDs was largely increased to 55 800 cd m^–2^, and the EQE was improved to 11.6%.^104^ Besides, MA-doped CsPbBr_3_ LEDs also gradually exhibit improved performance.^105^ Notably, Silver (Ag) in the cathode can be used to passivate the surface defects of CsPbI_3_, and can also be used as a dopant to reduce the electron injection barrier in CsPbI_3_ PeLEDs.^106^ As shown in Figure 10c, the EQE of CsPbI_3_ PeLEDs was improved to 11.2%. As shown in Figure 10d, the “electron-only” and “hole-only” devices demonstrate that the electron transport properties of Ag-doped PeLEDs are increased due to Ag doping. In addition, doping by cations with a larger size is more favorable for iodide-based perovskites, which contributes to the fabrication of high-performance LED devices. Therefore, bulk A-site doping can efficiently optimize LED performance.

Potassium (K) doping in CsPbX_3_ nanocrystals has been achieved by employing Cs_2_CO_3_, PbX_2_, and KX as precursor materials. Additionally, rubidium (Rb) doping has been accomplished in bulk Rb_*x*_Cs_1–x_PbCl_3_ and Rb_*x*_Cs_1–x_PbBr_3_ solid solutions through grinding and heating processes. Nanocrystals of Rb_*x*_Cs_1–x_PbCl_3_ and Rb_*x*_Cs_1–x_PbBr_3_ were synthesized using a hot-injection method. Colloidal Rb_*x*_Cs_1–x_PbBr_3_ nanocrystals exhibit green emission, reaching a maximum PLQY of approximately 60% for Rb_0.4_Cs_0.6_PbBr_3_. Remarkably, it has been shown that the emission wavelength can be adjusted from 460 to 500 nm while maintaining PLQYs greater than 60% through simple variations in reaction temperatures.^105,106^ Hence, all of these A-site dopants play a significant role in enhancing the performance of halide perovskite light-emitting diodes (PeLEDs).

## 5 Bandgap Control by B-Site Substitution

### 5.1 Impact of B-Site Cation Doping on Optoelectronic Properties of Perovskite NCs

B-site doping in perovskites significantly alters the electronic structure and properties of materials and is a critical area of study for enhancing the performance and stability of perovskite-based devices. B-site doping typically involves substituting the metal ion (commonly Pb^2+^ in halide perovskites) with other cations such as Sn^2+^, Mg^2+^, or transition metals such as Mn^2+^, Cu^2+^, or Fe^2+^. This substitution can significantly alter the bandgap of the perovskite. For instance, replacing lead with tin can reduce the bandgap, which is beneficial for applications that require absorption of lower-energy photons, such as in infrared photodetectors or solar cells targeting broader spectral absorption. Moreover, certain dopants can introduce midgap states that serve as recombination centers for electrons and holes, potentially improving the luminescence properties of LEDs. Doping with ions that have different ionic radii or charge states compared to lead can induce or relieve the lattice strain. This can enhance the mechanical stability of the perovskite crystal and make it less susceptible to thermal and mechanical degradation. For LED applications, B-site doping can be used to tune the emission wavelength and improve color purity by modifying the local electronic environment within the perovskite lattice. Furthermore, B-site dopants can stabilize the ionic lattice, reducing the propensity for ion migration under the device operating conditions. Certain metallic dopants can also improve the hydrophobicity of the perovskite lattice or lead to the formation of surface barriers that protect the sensitive underlying layers from moisture-induced degradation. Therefore, B-site doping plays a pivotal role in enhancing the functional properties of perovskites, addressing fundamental challenges related to their stability and performance and expanding their application scope in the field of advanced materials and devices.

Moreover, the influence of the B-site cation on the Goldschmidt tolerance factor (*t*) is particularly critical, as it can determine the overall stability and the ideal crystal structure of the unit cell.^80^ For a perovskite structure to be stable in the ideal cubic form, the tolerance factor should ideally be close to one. When the tolerance factor is around one, the octahedra formed by the X ions around the B-site are undistorted, leading to a stable perovskite structure. However, if the tolerance factor deviates significantly from one, the perovskite may adopt a noncubic structure (tetragonal, orthorhombic, etc.) or may even be unstable.^79^ For instance, when t is bigger than one, it indicates a larger A-site cation relative to the B-site, which can lead to a “stretched” octahedral cage, potentially causing the structure to become more open and possibly less stable.^85,86^ And when it is smaller than one, it suggests a smaller A-site cation relative to the B-site, leading to a “compressed” octahedral structure. This compression can destabilize the octahedra, often leading to tilting or distortion, which can impact the material’s electronic properties and mechanical stability.

**Figure 11:**
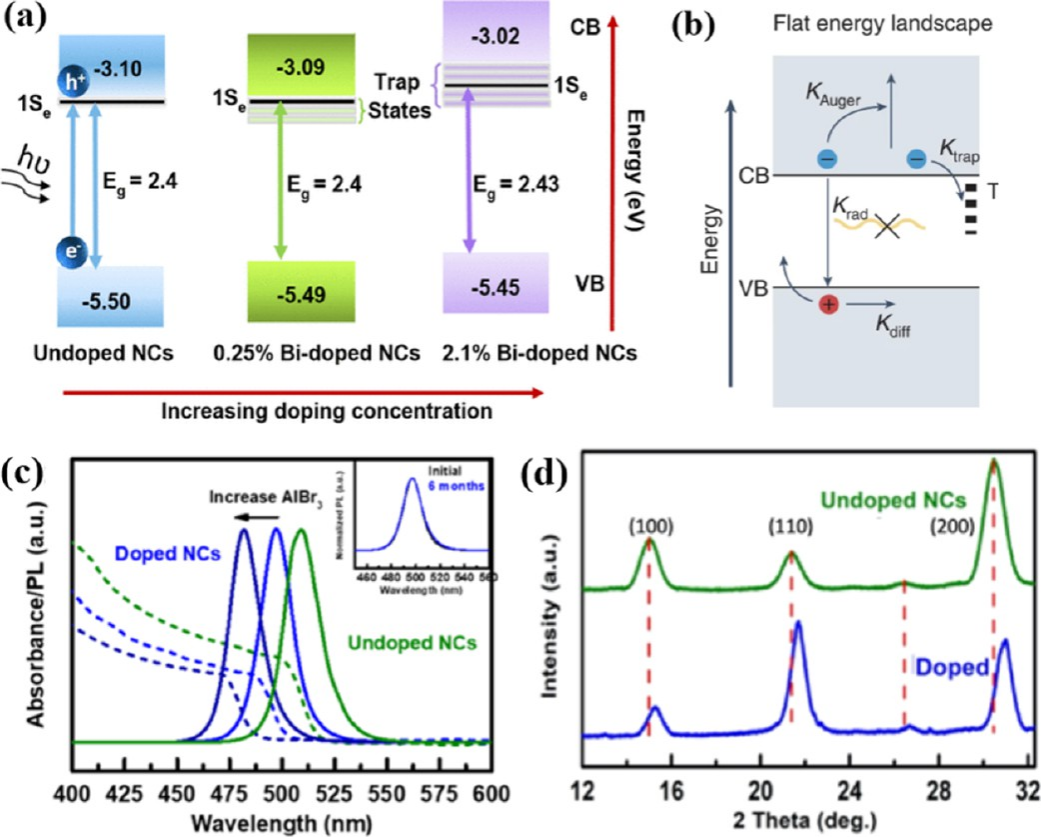
(a) Schematic representation showing changes in the band alignments of CsPbBr_3_ NCs upon doping with 0.25 or 2.1% Bi.^92^ (b) Radiative and nonradiative recombination pathways in 3D perovskites.^92^ Panels (a) and (b) are reproduced with permission from ref (92). Copyright 2017 American Chemical Society. (c) Absorption and PL spectra of undoped and doped NCs (Al/Pb ratio of 4.5 and 7.7), with the inset showing the PL of doped NCs (Al/Pb ratio 4.5) recorded directly after synthesis and after 6 months.^115^ (d) Zoom-in view of the X-ray diffractograms of doped and undoped CsPbBr NCs, highlighting the shift of the diffraction peaks upon Al_3_^+^ doping (Al/Pb input ratio: 4.5).^115^ Panels (c) and (d) are reproduced with permission from ref (115). Copyright 2022 American Chemical Society.

Numerous experiments have explored the incorporation of various metal ions into lead-halide perovskites (LHP). These ions can be categorized into two groups: divalent (Sn^2+^, Cd^2+^, Zn^2+^, Sr^2+^, and Mn^2+^), and trivalent (Al^3+^, RE^3+^, and Bi^3+^). Divalent ion-doped perovskites have been found to exhibit a wider optical bandgap, resulting in an absorption blue shift compared to their undoped counterparts.^48,107−109^ Doping with divalent ions, which are smaller in size than Pb^2+^, can lead to a reduction in the lattice size, resulting in shorter Pb–X bonds and an increased interaction between Pb and X orbitals. This interaction causes wider bandgaps in the CsPbX_3_ nanocrystals. However, in the case of Ni^2+^ doping, a red-shift is observed due to increased lattice order after the incorporation of Ni ions.^107^ Trivalent ions can alter the bandgap of LHP nanocrystals, impacting their optical properties, including the bandgap. The extent of this effect depends on the specific trivalent ion and its concentration in the nanocrystals.^59,110−92^ As shown in Figure 11a, the introduction of trivalent ions like Bi^3+^ or rare earth ions (e.g., Tm^3+^, Dy^3+^, Sm^3+^, Ce^3+^, Er^3+^, Yb^3+^, Eu^3+^) into LHP nanocrystals can influence their conduction bands and bandgaps. The specific effect is dependent on the type and concentration of the dopant. Additionally, B-site doping can modify the bandgap level by altering the surface defect state of LHP nanocrystals.^92^ In 3D perovskites, slow free electron–hole bimolecular radiative recombination (as shown in Figure 11b) presents a fundamental challenge for increasing PLQY and external quantum efficiency (EQE).^92^ This radiative recombination process competes with trap-assisted recombination at low excitation levels and with Auger recombination at high excitation levels. Quasi-2D perovskites, with the formula L_2_[ABX_3_]_(*n*−1)_BX_4_, feature a quantum well (QW) structure and also exhibit high radiative recombination due to exciton confinement.^114^

Recently, as shown in Figure 11c,d, Reiss and colleagues conducted room temperature doping of CsPbBr_3_ nanocrystals with Al^3+^ ions by immersing them in a solution of AlBr_3_ in dibromomethane. This doping process resulted in the tuning of the emission wavelength within the range of 510 to 480 nm, and the emission remained stable over time. The XRD image of both doped and undoped CsPbBr_3_ nanocrystals, highlighting the shift of the diffraction peaks upon Al^3+^ doping (with an Al/Pb input ratio of 4.5), is illustrated in Figure 11d. Furthermore, the PLQY of the as-synthesized CsPbBr_3_ nanocrystals was initially 72.5% and exhibited a slight decrease upon doping to 57.4 and 63.5% with Al/Pb ratios of 4.5 and 7.5, respectively.^115^

Except the bandgap tuning, B-site doping in perovskite nanocrystals has a significant influence on the optical properties of materials. The emission properties of perovskite nanocrystals can be tailored by B-site doping, which is crucial for developing LEDs with specific color outputs. Because the changes in the bandgap can directly translate to shifts in the absorption and emission spectra. For example, a narrower bandgap due to a larger B-site ion might result in emission at longer wavelengths (red-shift), while a wider bandgap due to a smaller B-site ion might cause blue-shifted emission. Moreover, the emission intensity and color purity can also be affected by B-site doping. Some dopants can introduce localized states within the bandgap that serve as new radiative recombination centers, enhancing emission intensity and color purity. Besides, the introduction of B-site dopants can significantly impact the stability of the photoluminescence. For example, certain dopants can passivate electronic traps within the crystal lattice that typically quench photoluminescence. By filling these traps, the dopants can reduce the nonradiative recombination pathways, enhancing the overall photoluminescence stability and efficiency. Dopants that improve the crystal’s structural integrity can also enhance its thermal stability, preserving photoluminescence characteristics under varied thermal conditions. Furthermore, B-site doping can alter the dynamics of charge carriers within the material, affecting both electron and hole mobilities. By modification of the lattice structure and the electronic environment, B-site dopants can influence the mobility of charge carriers, potentially improving the radiative recombination rates and efficiencies of devices like solar cells and LEDs. In conclusion, B-site doping in perovskite nanocrystals offers a versatile approach to tuning their optical and electronic properties. This technique allows for the precise control of emission characteristics, enhances photoluminescence stability, and can improve the efficiency of devices by optimizing the charge carrier dynamics and energy transfer processes. Such modifications are essential for tailoring perovskites to specific applications in the LEDs.

### 5.2 Impact of B-Site Doping on Perovskite LED Performance

**Figure 12:**
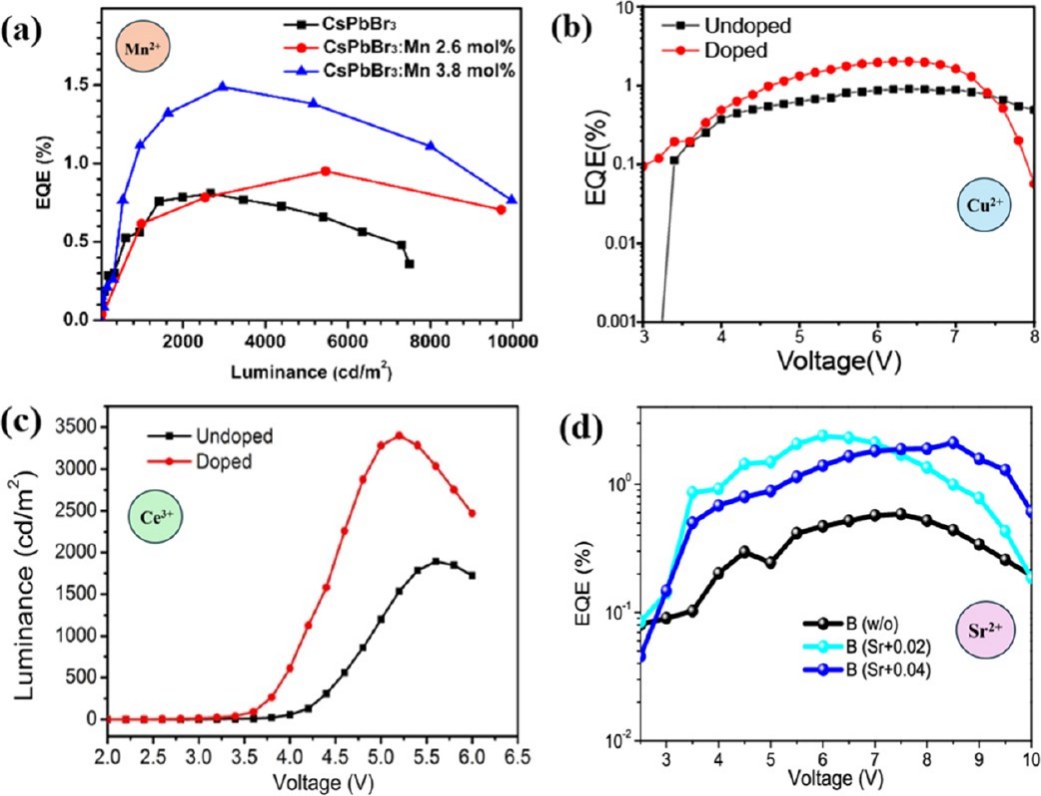
(a) EQE vs luminance of the LED device based on different doping concentrations.^37^ Reproduced with permission from ref (37). Copyright 2017 American Chemical Society. (b) EQE vs voltage curve of the PeLED device;^116^ Reproduced with permission from ref (116). Copyright 2021 American Chemical Society. (c) Luminance vs voltage of original CsPbBr_3_ and Ce^3+^-doped CsPbBr_3_-based LED device.^75^ Reproduced with permission from ref (75). Copyright 2018 American Chemical Society. (d) EQE vs voltage curve of the PeLED device with different concentrations of Strontium doping.^117^ Reproduced with permission from ref (117). Copyright 2023 American Chemical Society.

Numerous experiments have explored the incorporation of various B-site metal ions into perovskite quantum dots, primarily focusing on their photoluminescence properties. However, only a limited number of these studies have successfully translated these findings into practical applications in LEDs.^37^ It appears that Mn^2+^ doping can reduce energy transfer within CsPbCl_3_ nanocrystals, but this effect is not as significant when adding Mn^2+^ into CsPbBr_3_ nanocrystals.^37^ As shown in Figure 12a, the EQE of CsPbBr_3_ LEDs increases from 0.81 to 1.49% after Mn^2+^ doping and the EQE of Mn-doped CsPbI_3_ LEDs shows even significantly improvement.^37^

Replacing Pb with Sn^2+^ in CsPb_1–*x*_Sn_*x*_Br_3_ NCs has been suggested as a kind of nonradiative Auger recombination to reduce the level of triplet formation. Therefore, according to Wang et al, LEDs based on CsPb_0.67_Sn_0.33_Br_3_ QDs show the highest current efficiency (CE), EQE, and brightness.^76^ Moreover, according to Zhuo et al, the maximum EQE of the Cu^2+^-doped PeLED is much higher (2.03%) than that of the undoped PeLED (0.9%), which is shown in Figure 12b.^116^ Cerium (Ce) doping is believed to lower the charge-injection barrier without introducing additional trap states, and then provide efficient, barrier-free charge injection into NC emitters.^75^ The charge-injection barrier can be reduced by Ce doping, which will not change the state of the trap. Therefore, sufficient current is injected into LED light emitters. As shown in Figure 12c, and mentioned above, Ce^3+^ doped CsPbX_3_-based LED devices emit much brighter light and have a lower start voltage of 2.5 V.^75^ Besides, the EQE and CE of Ce^3+^ doped-based LED devices are also enhanced compared with those of undoped LED devices. According to Rogach et al., after both Cl-passivated and Sr^2+^-doped, the EQE of CsPbI_3_-based LED can be improved to 13.5%.^109^ Moreover, “electron-only” and “hole-only” devices show the enhanced hole-transport performance of LHP LED devices, which balances the rate of electron and hole transport, leading to the improvement of the recombination procedure.^109^

Besides, the PeLEDs employing the B (Sr+0.02) perovskite layer show the maximum EQE of 2.37% at 6 V (Figure 12d), which is about 4 times that of the undoped device.^117^

In summary, B-site doping in LHP LEDs can reduce Auger recombination, resulting in reduced charge-injection barriers, improved carrier transport, and enhanced device performance.^109^ While perovskite-based LEDs have shown promise, there is still room for improvement in their performance, including PLQY and stability. Doping is one avenue researchers are exploring to enhance the efficiency and overall performance of quantum-dot LEDs (QLEDs).^118^ Blue perovskite-based LEDs have faced challenges due to their relatively poor photoluminescence efficiency. This is an area where further research and development are needed to achieve efficient blue emissions in perovskite-based LEDs. Doping and other strategies may be explored to address this issue.^119^ B-site doping appears to be a promising approach to enhance the performance of blue perovskite-based LEDs and has potential applications in white LEDs as well. This strategy can provide multiple luminescent centers within one material, which is advantageous for achieving versatile and efficient lighting devices. Further research in this direction may lead to improvements in LED technology.^118,119^Table 4 provides an overview of various B-site dopants and their effects on perovskite nanocrystals.^74^

**Table 4:** Summary of the Emission Peak Wavelength, Full Width at Half-Maximum (FWHM), and PLQY of Metal Halide Perovskite NCs with Different B-Site Dopants^74^

doped nanocrystal composition	emission peak (nm)	FWHM (nm)	PLQY (%)
CsPb_0.9_Sn_0.1_Br_3_	519	19	91.9
CsPb_0.7_Sn_0.3_Br_3_	516	28	62
CsPb_0.5_Sn_0.5_Br_3_	503	27	41
CsPb_0.3_Sn_0.7_Br_3_	501	30	30
CsPb_0.1_Sn_0.9_Br_3_	521	—	9.2
CsPb_0.97_Mn_0.03_CI_3_	396/569	—	5
CsPb_0.94_Mn_0.06_CI_3_	396/574	—	22
CsPb_0.87_Mn_0.13_CI_3_	396/575	—	43
CsPb_0.73_Mn_0.27_CI_3_	396/579	—	54
CsPb_0.62_Mn_0.38_CI_3_	396/582	—	36
CsPb_0.54_Mn_0.46_CI_3_	396/587	—	17
CsPb_0.7_Ce_0.3_Br_3_	516	∼25	52
CsPb_0.66_Ce_0.34_Br_3_	∼514	∼25	64
CsPb_0.65_Ce_0.35_Br_3_	∼512	∼25	50
CsPb_0.55_Ce_0.45_Br_3_	∼512	∼25	78
CsPb_0.26_Ce_0.74_Br_3_	510	∼25	89

## 6 Impact of the Device Architecture on Performance of Perovskite LEDs

As outlined in the previous sections, all-inorganic CsPbX_3_ PeNCs offer notable advantages over organic and organic–inorganic hybrid perovskites. These advantages include enhanced stability, high PLQY, narrow luminous line widths, a broad color gamut, and various other exceptional optoelectronic properties.^27,120−124^ However, despite their impressive optoelectronic properties, CsPbX_3_ PeNCs are sensitive to high temperatures and humidity, which can limit their performance and long-term stability in photovoltaic devices. This sensitivity to environmental factors is one of the challenges that researchers are working to address in order to realize the full potential of these materials in various applications.^125^ When considering PeLEDs for LED applications, the primary types include phosphorescent conversion white LEDs (pc-white-LEDs) and electrically driven LEDs.^126^

Pc-white-LEDs, as advanced solid-state lighting (SSL) devices, have garnered significant interest due to their potential to greatly reduce energy consumption and greenhouse gas emissions in lighting.^127^ The main emission source in pc-white-LEDs is GaN or InGaN semiconductor chips, which emit near-ultraviolet light. Perovskite nanocrystals absorb a portion of this radiant light, down-convert it, and re-emit it across the visible spectrum.^128^ Yoon et al. developed a six-color display system using pc-white-LEDs that accurately replicates a realistic spectral distribution. They achieved this using pure-colored CsPbX_3_ (X = Cl, Br, I, or Cl/Br and Br/I) based on monochrome down-conversion LEDs using perovskite nanocrystals.^129,130^ The produced LED demonstrates moderate luminous efficiency at 62 lm/W with a total current of 120 mA. It also achieves excellent color quality, including a high color rendering index (CRI) of 96 and a red special CRI of 97. This suggests the feasibility of creating a color-by-blue backlight display for future field sequential color liquid crystal LEDs with outstanding visual and color performance.

Another solid-state lighting (SSL) device that utilizes CsPbX_3_ PeNCs is an electrically driven LED. This LED is designed with a double heterojunction structure, featuring an intrinsic active layer placed between an n-type electron transport layer and a p-type hole-transport layer. When a forward bias is applied, charge carriers are injected into the perovskite layer. Within this layer, they undergo radiative recombination, resulting in light emission in all directions.^128−132^ The LED device is composed of several layers, including an anode, a buffer layer, a hole-transport layer, a perovskite film, another hole-transport layer, and a cathode. In the case of CsPbX_3_ PeLEDs, significant challenges and opportunities for improvement remain in terms of efficiency. Thin-film perovskite devices currently face efficiency limitations due to issues related to surface passivation and film-forming properties, making them less efficient than bulk perovskite counterparts.^133^

**Figure 13:**
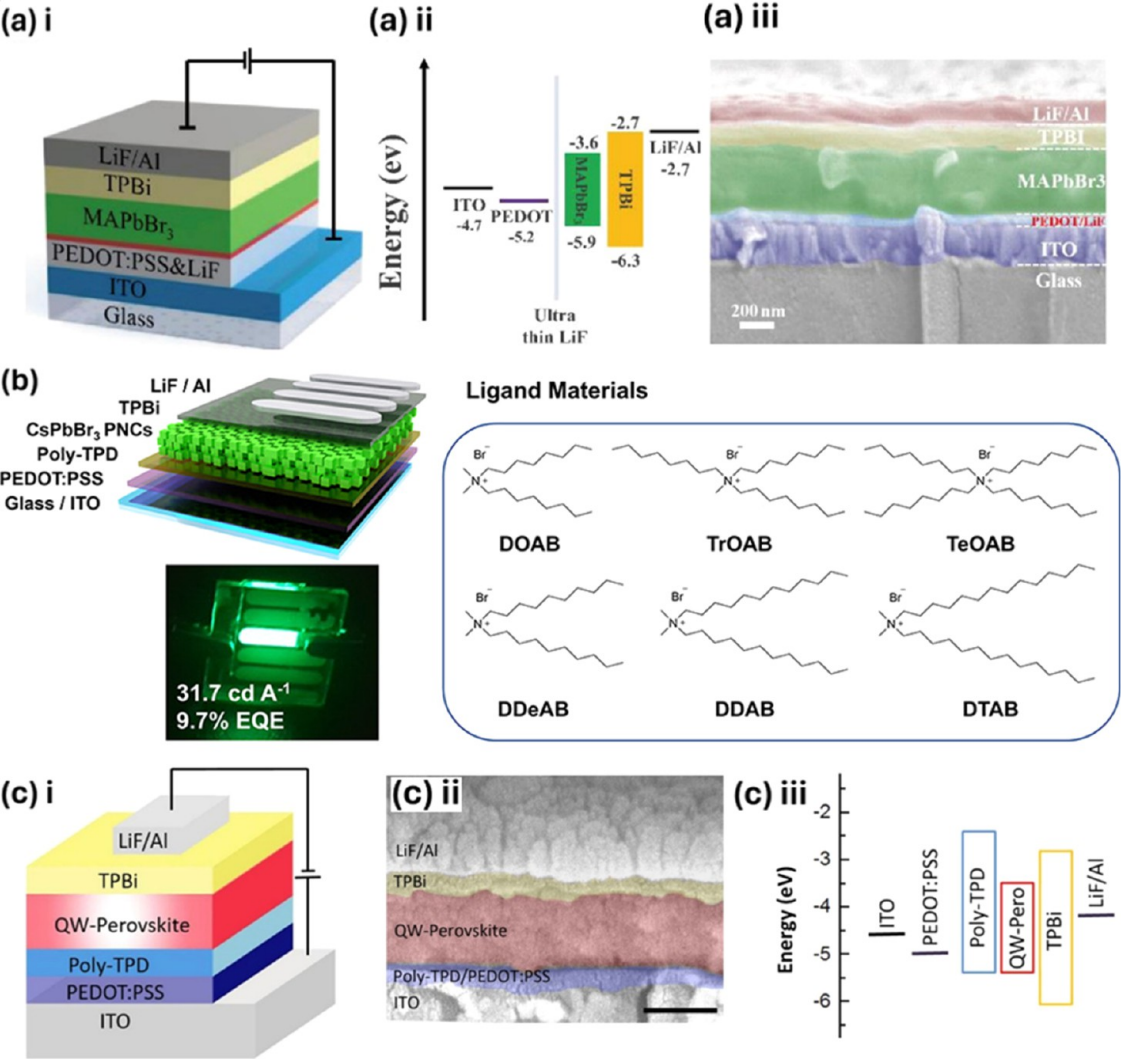
Illustration of a multilayer perovskite quantum light-emitting device. (a) i ITO/PEDOT:PSS/PVK/QDs/TPBi/LiF/Al structure; (a) ii energy level distribution diagram of PeLED; (a) iii TEM image showing the cross-section of a multilayer material modified with 2 nm LiF.^134^ Panels (a) i, (a) ii, and (a) iii are reproduced with permission from ref (134). Copyright 2020 Organic Electronics. (b) Structure of the green PeLED.^135^ Reproduced with permission from ref (135). Copyright 2019 American Chemical Society. (c) i Schematic of the device structure and (c) ii their SEM image; and (c) iii energy levels of the corresponding layers in perovskite LEDs.^136^ Panels (c)i, (c) ii, and (c) iii are reproduced with permission from ref (136). Copyright 2016 Scientific Reports.

Zeng et al. were the first to create an electrically driven LED by using CsPbX_3_ NCs. The perovskite layer was made from pure CsPbX_3_ NCs synthesized by a hot-injection method. The LED structure included layers arranged in the following order: indium tin oxide (ITO), poly(ethylenedioxythiophene): polystyrene sulfonate (PEDOT: PSS, 40 nm), poly(9-vinylcarbazole) (PVK, 10 nm), perovskite (10 nm), TPBi (40 nm), and LiF/Al (1/100 nm).^133^ These LEDs emitted blue, green, and orange light with brightness levels of 742, 946, and 528 cd/m^2^, respectively. They achieved EQE values of 0.07, 0.12, and 0.09%, respectively.^133^ The classic device structure (ITO/PEDOT/LiF/perovskite/TPBi/LiF/Al) and energy level distribution diagram of PeLED are shown in Figure 13(a)i and ii respectively.^134^Figure 13(a)iii is a cross-sectional scanning electron microscopy (SEM) image of PeLED modified with 2 nm LiF.^134^

Subsequently, various optimization methods have been investigated to enhance the photovoltaic performance of electrically driven LEDs utilizing CsPbX_3_ NCs. The primary optimization approaches focus on doping, cross-linking, and surface passivation of the perovskite layer, as well as optimizing the interface between the electron transport layer and the hole-transport layer with the emission layer.^133^ As shown in Figure 13b, Jong et al. achieved a high-efficiency green LED with a current efficiency of 31.7 cd A^–1^ and an EQE of 9.7%, which was achieved by the optimized didecyldimethylammonium bromide ligands.^135^ Moreover, Li et al. introduced a novel cross-linking technique utilizing trimethylaluminum (TMA) vapor, resulting in nearly complete coverage of nanocrystalline films. The process involves depositing a ZnO nanocrystalline film directly onto an ITO-coated glass substrate, followed by deposition of the perovskite layer as the emission layer. However, the subsequent solution deposition of the charge-injection layer is restricted due to the solubility of the perovskite film in organic solvents.^137−140^ The innovative TMA vapor-phase cross-linking method is implemented by subjecting the perovskite film to a brief exposure to TMA vapor, followed by placing the treated film in ambient air.^23^ This cross-linking technique renders the CsPbX_3_ NCs film insoluble, facilitating the deposition of a layer of TFB polymer (poly[(9,9-dioctylfluorenyl-2,7-diyl)-*co*-(4,4′-(*N*-(4-s-butylphenyl)diphenylamine)]). Ultimately, the LED device is constructed using an ITO, ZnO, TMA-treated CsPbI_3_, TFB, and MoO_3_/Ag structure, achieving an EQE as high as 5.7%.^141^

According to Hu et al., deep saturated red emission was obtained with a peak QEQ of 2.29% and a maximum luminance of 214 cd/m^2^.^136^ The quantum well perovskite LEDs with a multilayered structure are shown in Figure 13(c)i and ii. Figure 13(c)iii shows a schematic of the energy level diagram of all the layers.^136^ Moreover, Zeng and colleagues optimized CsPbX_3_ QLEDs by achieving an efficient solution-processed CsPbBr_3_ QLED. They achieved this by carefully balancing surface passivation and carrier injection through precise control of ligand density.^139,142−145^ They introduced a novel approach using a mixed solvent of hexane and ethyl acetate to recycle quantum dots and control the surface ligand density. Additionally, they improved the hole-transport layer and selected more effective solvents as part of their optimization efforts.^146^ PolyTPD is employed as a hole-transport layer instead of poly(9-vinylcarbazole) (PVK), and they used ethyl acetate as a processing solvent instead of acetone.^147,148^ After various improvements, the EQE of QLED devices increased from 0.12 to 6.27% (Figure 13c).^147^ Up to now, improving LED efficiency has been a prominent research focus, and the EQE of all-inorganic PeLEDs has surpassed 15%. The specific developments in the optical performance are summarized in Table 5.

**Table 5:** Summary of the Different Architectures and the Optical Performance of LEDs Using CsPbX_3_ NCs and Doped Systems as the Light-Emitting Layer

emitter composition	LED structure	EL λ_max_ (nm)	*V*_on_ (V)	EQE (%)	*L*_max_ (cd m^–2^)	year	ref
CsPbBr_3_	ITO/PEDOT:PSS/poly-TPD/perovskite/TPBi/LiF/Al	516	3.5	0.06	1377	2016	(149)
CsPbBr_3_	ITO/ZnO/perovskite/TFB/MoO_3_/Ag	523	2.8	0.19	2333	2016	(141)
CsPbI_3_	ITO/ZnO/perovskite/TFB/MoO_3_/Ag	698	2.2	5.7	206	2016	(141)
CsPbI_2.25_Br_0.75_	ITO/ZnO/perovskite/TFB/MoO_3_/Ag	619	/	1.4	2335	2016	(141)
CsPbI_1.5_Br_1.5_	ITO/ZnO/perovskite/TFB/MoO_3_/Ag	480	/	0.0074	8.7	2016	(141)
CsPbBr_3_	ITO/PEDOT: PSS/perovskite films/T8/Ca/Ag	528	3	0.035	407	2015	(150)
CsPbBr_3_	ITO/PEDOT:PSS/perovskite/TPBi/LiF/Al	527	4.6	2.21	3853	2016	(151)
CsPbBr_3_	ITO/PEDOT:PSS/polyTPD/perovskite/TPBi/LiF/Al	512	3.4	6.27	1518	2017	(144)
CsPb(Cl/Br)_3_	ITO/PEDOT:PSS/polyTPD/perovskite/TPBi/LiF/Al	455	5.1	0.07	742	2015	(133)
CsPbBr_3_	ITO/PEDOT:PSS/polyTPD/perovskite/TPBi/LiF/Al	516	4.2	0.12	946	2015	(133)
CsPb(Br/I)_3_	ITO/PEDOT:PSS/polyTPD/perovskite/TPBi/LiF/Al	586	4.6	0.09	528	2015	(133)
CsPbCl_1.7_Br_1.3_:Ni	ITO/PEDOT:PSS/polyTPD/perovskite/TPBi/LiF/Al	460	3.8	1.35	33	2019	(152)
CsPbBr_3_:Rb	ITO/PEDOT:PSS/polyTPD/perovskite/TPBi/LiF/Al	464	/	0.11	71	2019	(152)
CsPbBr_3_:Rb	ITO/PEDOT:PSS/polyTPD/perovskite/TPBi/LiF/Al	490	/	0.87	186	2019	(152)
CsPb(ClBr)_3_:Mn	ITO/PEDOT/TFB:PFI/perovskite/TPBi/LiF/Al	466	1	2.12	245	2018	(153)
CsPb(ClBr)_3_:Ni	ITO/PEDOT: PSS/TFB:PFI/perovskite/TPBi/LiF/Al	460	3.2	2.4	612	2020	(154)
CsPbBr_3_:Sn	ITO/PEDOT:PSS/polyTPD/perovskite/TPBi/LiF/Al	508	5	3.6	5495	2016	(41)
CsPbBr_3_:Ce	ITO/PEDOT:PSS/polyTPD/perovskite/TPBi/LiF/Al	510	3.8	4.4	/	2018	(75)
CsPbBr_3_:Mn	ITO/PEDOT:PSS/polyTPD/perovskite/TPBi/LiF/Al	511	4.2	1.49	9971	2017	(37)
CsPbBr_3_:Sn	ITO/PEDOTPSS/TFB/perovskite/TPBi/LiF/Al	517	3.6	4.13	12500	2017	(76)
CsPbBrI_2_:Cu	ITO/ZnO/PEI/perovskite/TCTA/MoO_3_/Al	630	2.2	5.1	/	2019	(156)
CsPbI_3_:Zn	ITO/PEDOT:PSS/TPAA/perovskite/TPBi/LiF/Al	687	/	14.6	378	2019	(55)
CsPbI_3_:Sr	ITO/PEDOT:PSS/polyTPD/perovskite/TPBi/LiF/Al	678	3.6	5.92	1250	2018	(106)
CsPbI_3_:Mn	ITO/PEDOT:PSS/polyTPD/perovskite/TPBi/LiF/Al	685	4.1	1.04	132	2017	(37)
CsPbI_3_:Ag	ITO/ZnO/PEI/perovskite/TCTA/MoO_3_/Au	690	/	11.2	1106	2018	(106)
CsPbI_3_:Sr	ITO/ZnO/PEI/perovskite/TCTA/MoO_3_/Au	691	2.0	13.5	1152	2018	(109)

## 7 Conclusions and Outlook

Emerging perovskite nanocrystals have demonstrated significant potential for use in a wide range of optoelectronic devices due to their outstanding optoelectronic and photophysical properties, as well as the cost-effective and straightforward synthetic methods available for their production. While there have been significant advancements in the development of new synthetic methods for perovskite nanocrystals, their susceptibility to environmental factors such as humidity and light remains a major challenge. This limitation significantly restricts their potential applications and future commercialization. In order to improve the luminescence properties and operational stability under harsh environmental conditions, one of the potential strategies is to develop a core. Therefore, this architecture represents a promising avenue to alleviate stability issues and thus drive improvements in the operational stability and performance of devices.

Understanding the mechanisms of doping and ion substitution in perovskite nanocrystals is still a complex and ongoing research challenge. It is difficult to precisely determine the location and distribution of dopants within the host matrix. To address this, researchers employ a combination of techniques such as X-ray photoelectron spectroscopy (XPS),^157^ extended X-ray absorption fine structure (EXAFS) spectroscopy,^158^ high-resolution synchrotron XRD,^157,159^ and first-principles calculations. These methods are used to investigate the doping mechanisms and accurately identify the positions of dopant ions within the lattice, which are crucial for drawing accurate conclusions. Optical spectroscopy (UV–visible absorption spectroscopy, PL spectroscopy, and transient absorption spectroscopy) plays an important role in investigating doping mechanisms. UV–visible absorption spectroscopy is used to determine the optical bandgap of perovskites, which is critical for applications like solar cells and LEDs where specific bandgap values are required for efficient operation.^160^ PL spectroscopy is crucial for evaluating the quality of the perovskite, including the purity of its emission and the presence of nonradiative recombination pathways, which affect the efficiency of light-emitting devices. Transient absorption spectroscopy is useful for understanding charge carrier dynamics, such as how quickly and efficiently carriers are generated, transported, and recombined, which is vital for optimizing device performance.^160^ Therefore, optical spectroscopy can be widely used in bandgap engineering, defect analysis, device optimization, and stability testing.^160^ Electron paramagnetic resonance (EPR) is also a powerful spectroscopy technique used to study materials with unpaired electrons in perovskite. It can provide quantitative information about the concentration of paramagnetic defects within perovskite materials.^161^

Moreover, ultrafast femtosecond transient absorption (fs-TA) spectroscopy can be used in the measurement of carrier dynamics, monitoring of photoinduced changes, detection of intermediate states, and study of phase segregation and degradation.^162^ It provides information about the lifetimes and mobility of charge carriers, which are crucial for understanding how they recombine to emit light in LEDs.^162^ Time-resolved photoluminescence (TRPL) spectroscopy provides insights into the photophysical dynamics of these materials by measuring the lifetime of photoluminescence after excitation.^163^ It can be used in the measurement of photoluminescence decay, identification of radiative and nonradiative processes, and determination of multiple decay channels. It provides detailed insights into the photophysical processes that directly impact the efficiency, stability, and overall performance of perovskite-based devices, thereby guiding the optimization of these materials for PeLEDs.^163^

When metal halide salts (such as SnCl_4_, VCl_3_, BiCl_3_, CuCl, PbCl_2_, NiCl_2_, and ZnCl_2_) are introduced as dopants to CsPbCl_3_ nanocrystals, the variations in the enhancement of PLQY are not solely attributed to the presence of these different metal ions. Instead, the differences arise from the diverse capacities of these metal salts to release active chloride ions for surface passivation. Control experiments with metal acetate salts, which do not increase PLQY, support this conclusion. Moreover, there is a significant knowledge gap within the research community concerning the impact of dopants on the operational stability of devices. Dopants often enhance the optical properties and, in some cases, improve the optical stability of nanocrystal solutions. It is important to acknowledge that dopants can potentially introduce instability under biased conditions. To gain a deeper understanding of this phenomenon, conducting systematic operando experiments on different types of doped perovskite nanocrystals is essential. These experiments will provide valuable insights into the effects of dopants on the stability of the materials under operational conditions. An additional unresolved question relates to the influence of dopants on the surface termination and passivation of perovskite nanocrystals, as well as their interaction with surface ligands like phosphonate ligands. Understanding these effects is crucial for elucidating the underlying mechanisms and optimizing the performance of doped perovskite nanocrystals in various applications. Further research is needed to explore these interactions and their impact on the properties and behavior of the nanocrystals.

Investigating the photoluminescence decay kinetics is crucial to understanding how surface defects, acting as recombination centers, impact the PLQY of perovskite nanocrystals. For example, a transition from biexponential PL decay to monoexponential decay indicates efficient passivation of surface defects. Robust techniques, such as transient absorption, time-resolved PL, and time-resolved fluorescence quenching spectroscopies, can be used to explore the energy transfer processes in perovskite nanocrystals. These techniques help examine exciton diffusion length and probe the energy transfer rate between neighboring nanocrystals. These fundamental studies are essential for assessing the suitability of nanocrystals for specific applications and may also aid in improving the PLQY of chloride-based and lead-free perovskite nanocrystals with low PLQY. Additionally, it is worth considering further research into the ferroelectric and piezoelectric properties of perovskite nanocrystals and their potential applications. Colloidal nanocrystals offer unique tunable physical properties through control over their size, shape, architecture (e.g., core–shell, nanowires, nanorods), and surface ligands. Future research directions may focus on advanced architectural engineering combined with composition tailoring. For example, exploring core–shell nanocrystals with one material doped in the core and another in the shell, or creating Janus structures with two different materials on each side, could lead to intriguing polarized optical or catalytic effects.^164,165^ According to Lin, the shell material can significantly improve the thermal stability of the core perovskite, which is particularly important for applications involving high operating temperatures or for processing techniques that involve thermal processing.^165,166^ Moreover, fine-tuning doping conditions to enhance efficiency and leveraging doping and ion substitution to modify optoelectronic properties, improve stability, and reduce toxicity make metal halide perovskite nanocrystals promising for various applications.

The remarkable optical properties of all-inorganic perovskite nanocrystals hold great promise for various applications including photodetectors, solar cells, lasers, and LEDs. Although significant progress has been made, this field is still in its early stages, and several challenges remain to be addressed. These challenges include improving the stability, reducing toxicity, and developing simpler synthesis methods. The lower stability of these nanocrystals has limited their commercial applications, and novel approaches are needed to overcome this limitation. Additionally, there is potential to explore other applications such as light-induced polymerization, photocatalysis, and anticounterfeiting using all-inorganic perovskite nanocrystals. While introducing these nanocrystals into these applications poses challenges, it is an area worth further exploration. In summary, despite the existing challenges, significant research progress has been made in the field of all-inorganic perovskite nanocrystals. Further research and exploration hold great potential for the development of innovative technologies and applications in the future.
